# Supplementary material for: Intermediately synchronised brain states optimise trade-off between subject specificity and predictive capacity
Source: Commun Biol. 2023 Jul 10;6:705. doi: 10.1038/s42003-023-05073-w (PMC10333234; doi:10.1038/s42003-023-05073-w)
Supplement: Supplementary file 2 — Supplementary Information [file 42003_2023_5073_MOESM2_ESM.pdf]

Intermediately Synchronised Brain States Optimise Trade-off between Subject Specificity and Predictive Capacity

Supplementary Information

Leonard Sasse[1, 2, 3], Daouia I. Larabi[1, 2], Amir Omidvarnia[1, 2], Kyesam Jung[1, 2], Felix Hoffstaedter[1, 2], Gerhard Jocham[4], Simon B. Eickhoff[1, 2], and Kaustubh R. Patil *[1, 2]

[1]Institute of Neuroscience and Medicine, Brain and Behaviour (INM-7), Research Centre Jülich, Jülich, Germany
[2]Institute of Systems Neuroscience, Medical Faculty, Heinrich-Heine-University Düsseldorf, Düsseldorf, Germany
[3]Max Planck School of Cognition, Stephanstrasse 1a, Leipzig, Germany
[4]Institute for Experimental Psychology, Faculty of Mathematics and Natural Sciences, Heinrich-Heine-University Düsseldorf, Düsseldorf, Germany

June 22, 2023

*k.patil@fz-juelich.de

Supplementary Table 1. Behavioural prediction targets from the **HCP-YA** sample

Name	HCP-YA field	Category
Visual Episodic Memory	PicSeq_Unadj	Cognition
Cognitive flexbility (DCCS)	CardSort_Unadj	Cognition
Inhibition (Flanker Task)	Flanker_Unadj	Cognition
Fluid Intelligence (PMAT)	PMAT24_A_CR	Cognition
Reading (pronounciation)	ReadEng_Unadj	Cognition
Vocabulary (picture matching)	PicVocab_Unadj	Cognition
Processing Speed	ProcSpeed_Unadj	Cognition
Delay Discounting	DDisc_AUC_40K	Cognition
Spatial Orientation	VSPLOT_TC	Cognition
Sustained Attention - Sens.	SCPT_SEN	Cognition
Sustained Attention - Spec.	SCPT_SPEC	Cognition
Verbal Episodic Memory	IWRD_TOT	Cognition
Working Memory (list sorting)	ListSort_Unadj	Cognition
Emotional Face Matching	Emotion_Task_Face_Acc	In-Scanner Task Performance
Arithmetic	Language_Task_Math_ Avg_Difficulty_Level	In-Scanner Task Performance
Story comprehension	Language_Task_Story_ Avg_Difficulty_Level	In-Scanner Task Performance
Relational processing	Relational_Task_Acc	In-Scanner Task Performance
Social Cognition - random	Social_Task_Perc_Random	In-Scanner Task Performance
Social Cognition - interaction	Social_Task_Perc_TOM	In-Scanner Task Performance
Working Memory (n-back)	WM_Task_Acc	In-Scanner Task Performance
Argreeableness (NEO)	NEOFAC_A	Personality
Openness (NEO)	NEOFAC_O	Personality
Conscientiousness (NEO)	NEOFAC_C	Personality
Neuroticism (NEO)	NEOFAC_N	Personality
Extraversion (NEO)	NEOFAC_E	Personality

Supplementary Table 2. Behavioural prediction targets from the **HCP-A** sample

Name	NDA data structure	HCP-A field	category
Crystal. Cog. Comp. Score	cogcomp01	nih_crycogcomp_unadjusted	Cognition
Lang./Vocab. Comprehension	tpvt01	tpvt_uss	Cognition
Cog. Flexibility	dccs01	nih_dccs_unadjusted	Cognition
Fluid Cog. Comp. Score	cogcomp01	nih_fluidcogcomp_unadjusted	Cognition

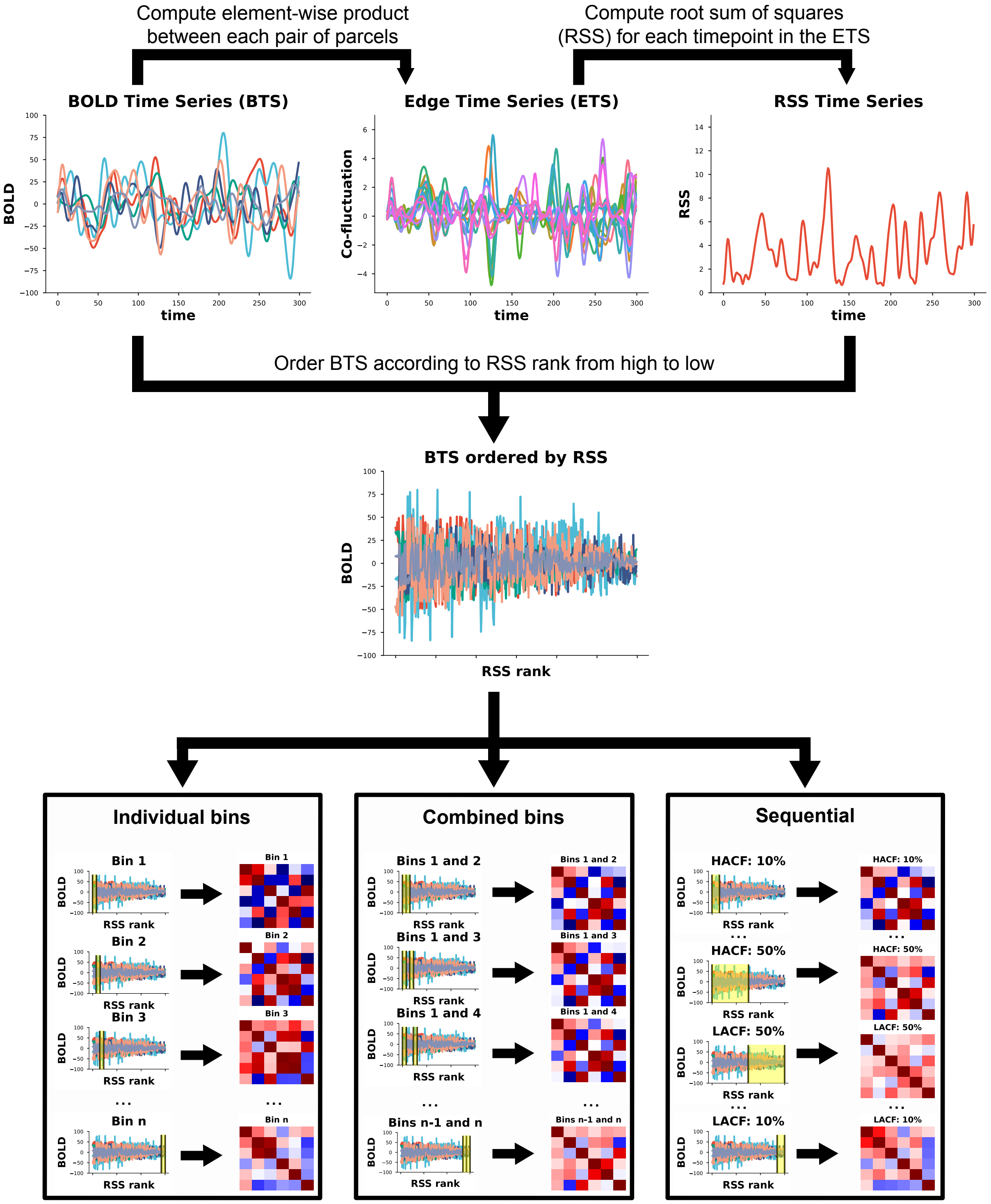

Supplementary Figure 1. Per subject workflow to extract FC at different levels of co-fluctuation using three different sampling strategies; **1)** in the ***individual bins strategy*** the re-ordered BOLD time series are divided into equally-sized bins, **2)**, in the ***combined bins strategy***, every possible combination of two bins was selected, frames from these two bins were concatenated, and **3)** in the ***sequential sampling strategy***, HACF or LACF frames were consecutively included to include only a percentage of the highest and lowest amplitude frames for each subject.

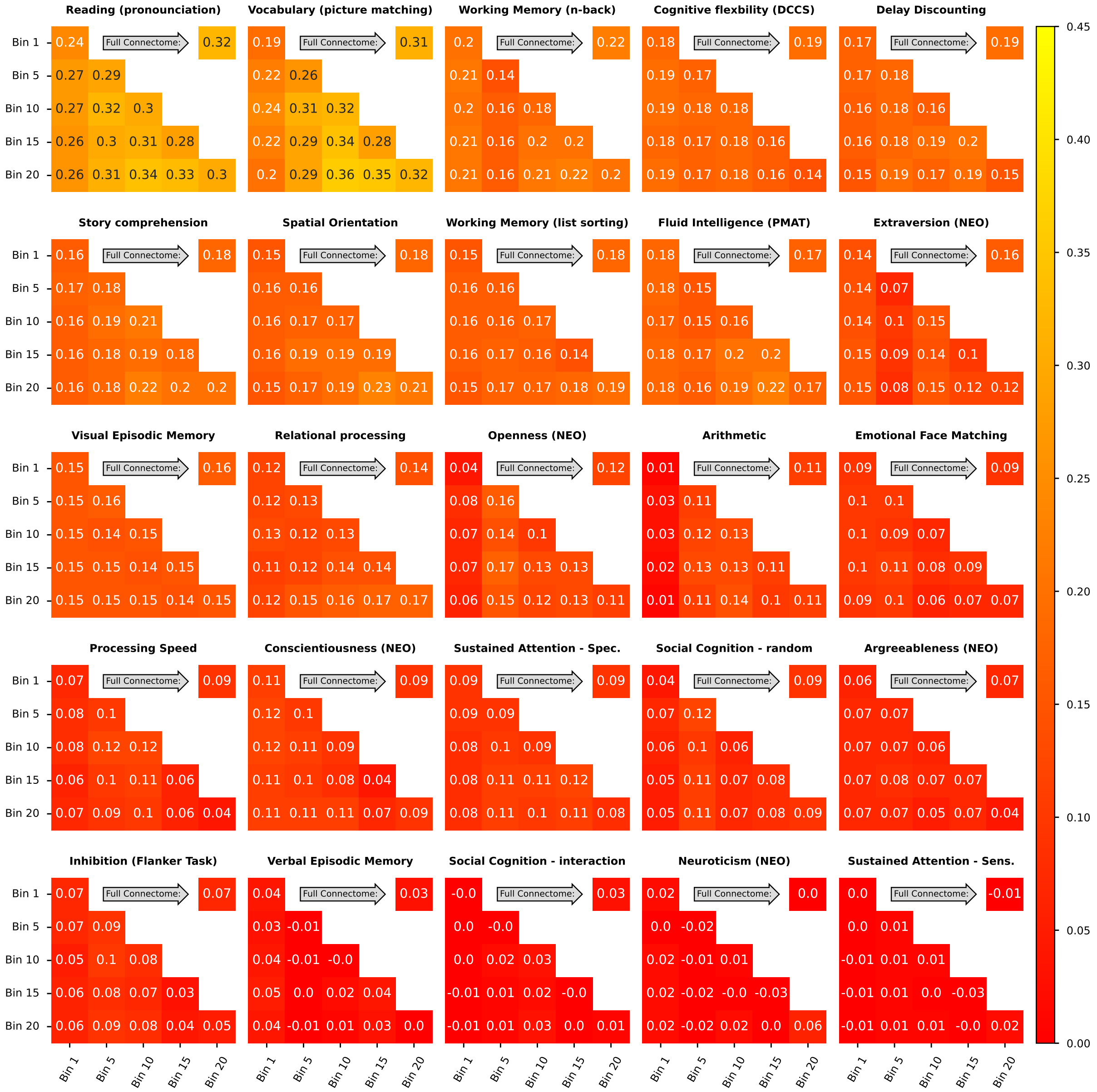

Supplementary Figure 2. Prediction scores (**Pearson's r** between observed and predicted values) in the **HCP-YA** sample for all targets using **kernel ridge regression** averaged across the ten folds in the grouped cross-validation scheme when using combined and individual bins sampling strategies and the **200 area Schaefer parcellation with global signal regression**. Scores for individual bins are displayed on the diagonal, for combined bins off the diagonal. Scores for the full FC using the whole time series are always displayed in the upper right corner.

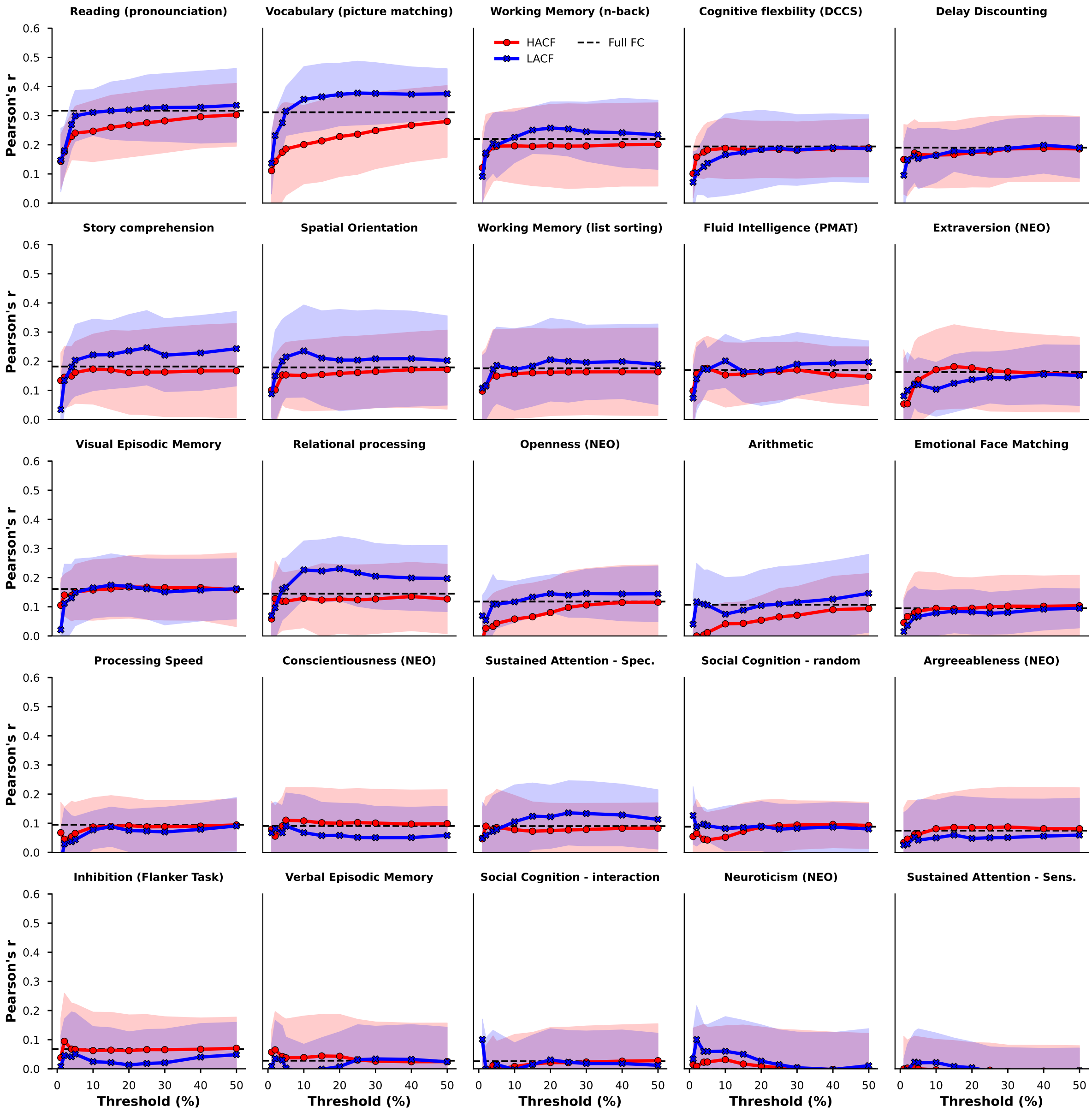

Supplementary Figure 3. Prediction scores (**Pearson's r** between observed and predicted values) in the **HCP-YA** sample for **kernel ridge regression** averaged across the ten folds in the grouped cross-validation scheme when using the **200 area Schaefer parcellation with global signal regression** and FC estimates derived from timepoints at different levels of co-fluctuation magnitude in the sequential sampling strategy.

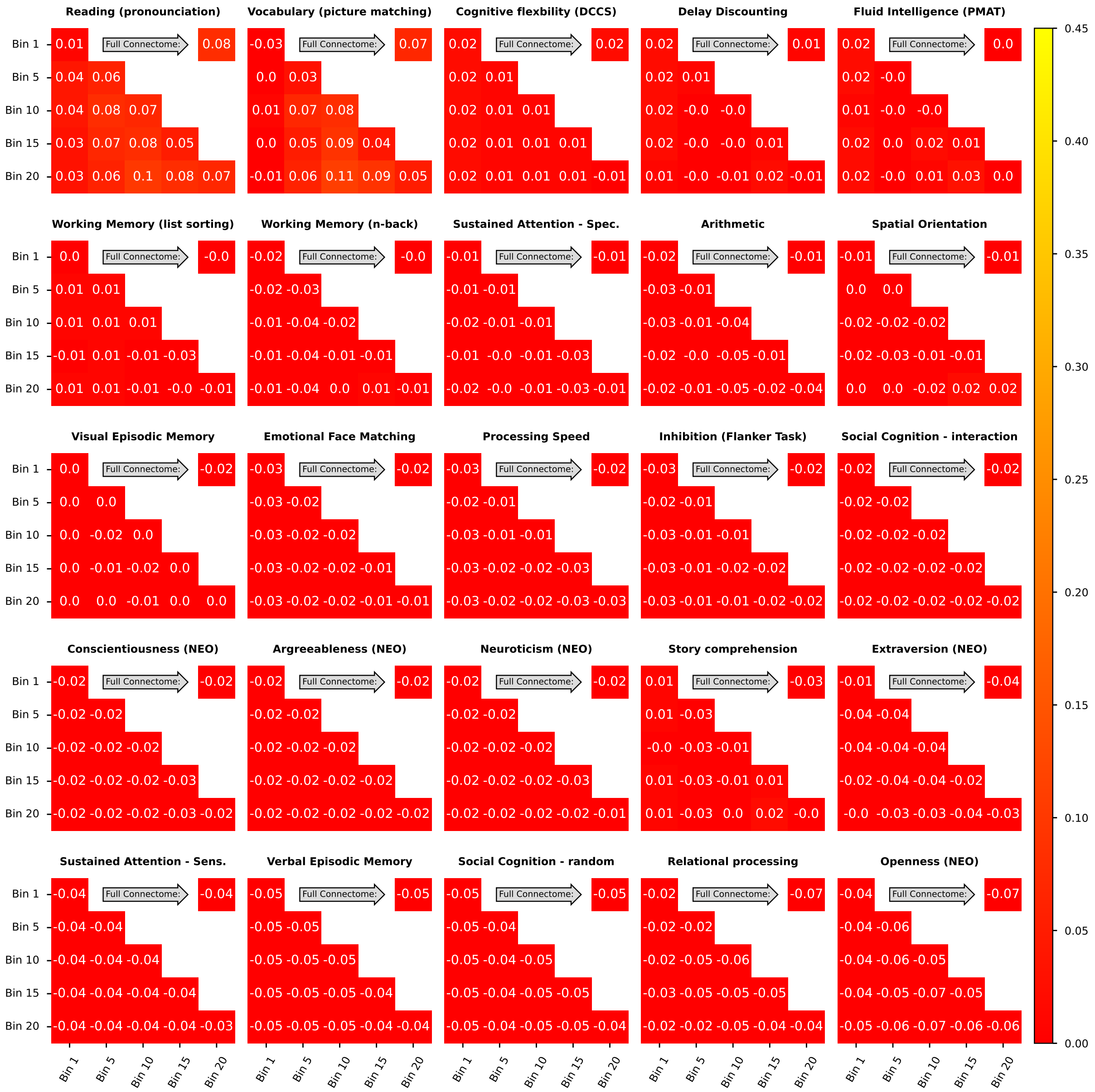

Supplementary Figure 4. Prediction scores (**r-squared**) in the **HCP-YA** sample for all targets using **kernel ridge regression** averaged across the ten folds in the grouped cross-validation scheme when using combined and individual bins sampling strategies and the **200 area Schaefer parcellation with global signal regression**. Scores for individual bins are displayed on the diagonal, for combined bins off the diagonal. Scores for the full FC using the whole time series are always displayed in the upper right corner.

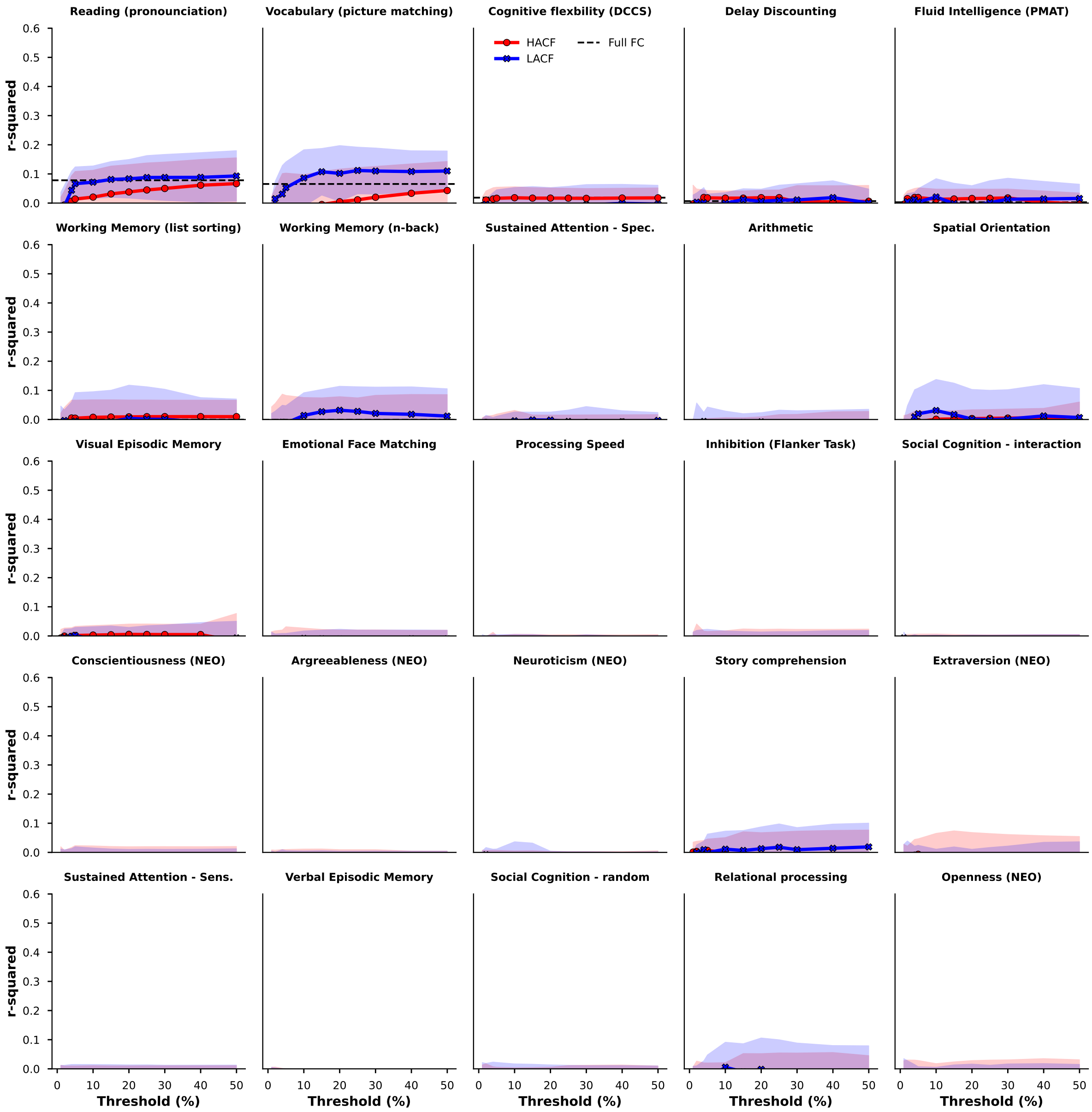

Supplementary Figure 5. Prediction scores (**r-squared** between observed and predicted values) in the **HCP-YA** sample for **kernel ridge regression** averaged across the ten folds in the grouped cross-validation scheme when using the **200 area Schaefer parcellation with global signal regression** and FC estimates derived from timepoints at different levels of co-fluctuation magnitude in the sequential sampling strategy.

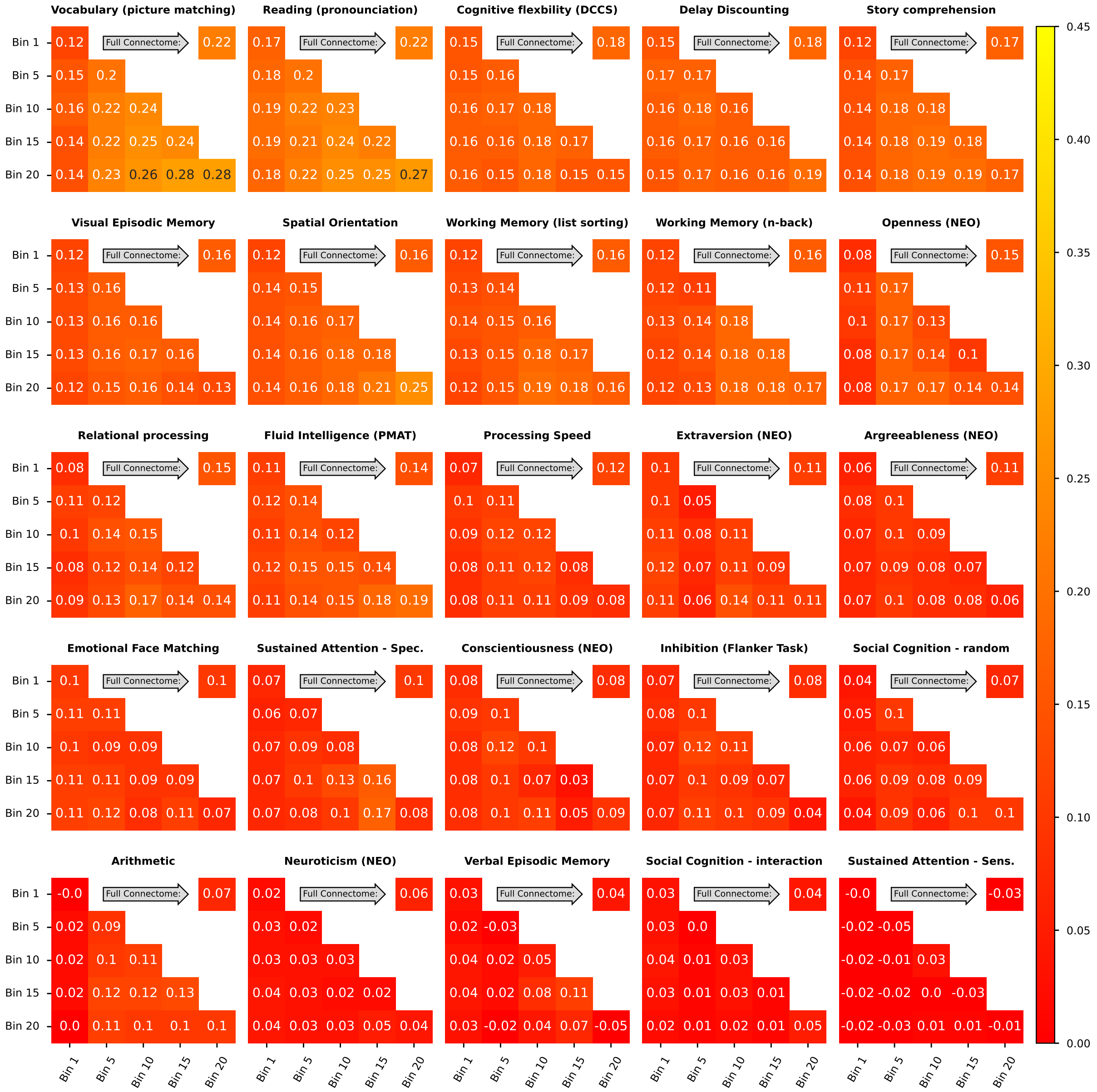

Supplementary Figure 6. Prediction scores (**Pearson's r** between observed and predicted values) in the **HCP-YA** sample for **Connectome-based Predictive Modeling (CBPM)** averaged across the ten folds in the grouped cross-validation scheme when using combined and individual bins sampling strategies and the **200 area Schaefer parcellation**. Scores for individual bins are displayed on the diagonal, for combined bins off the diagonal. Scores for the full FC using the whole time series are always displayed in the upper right corner.

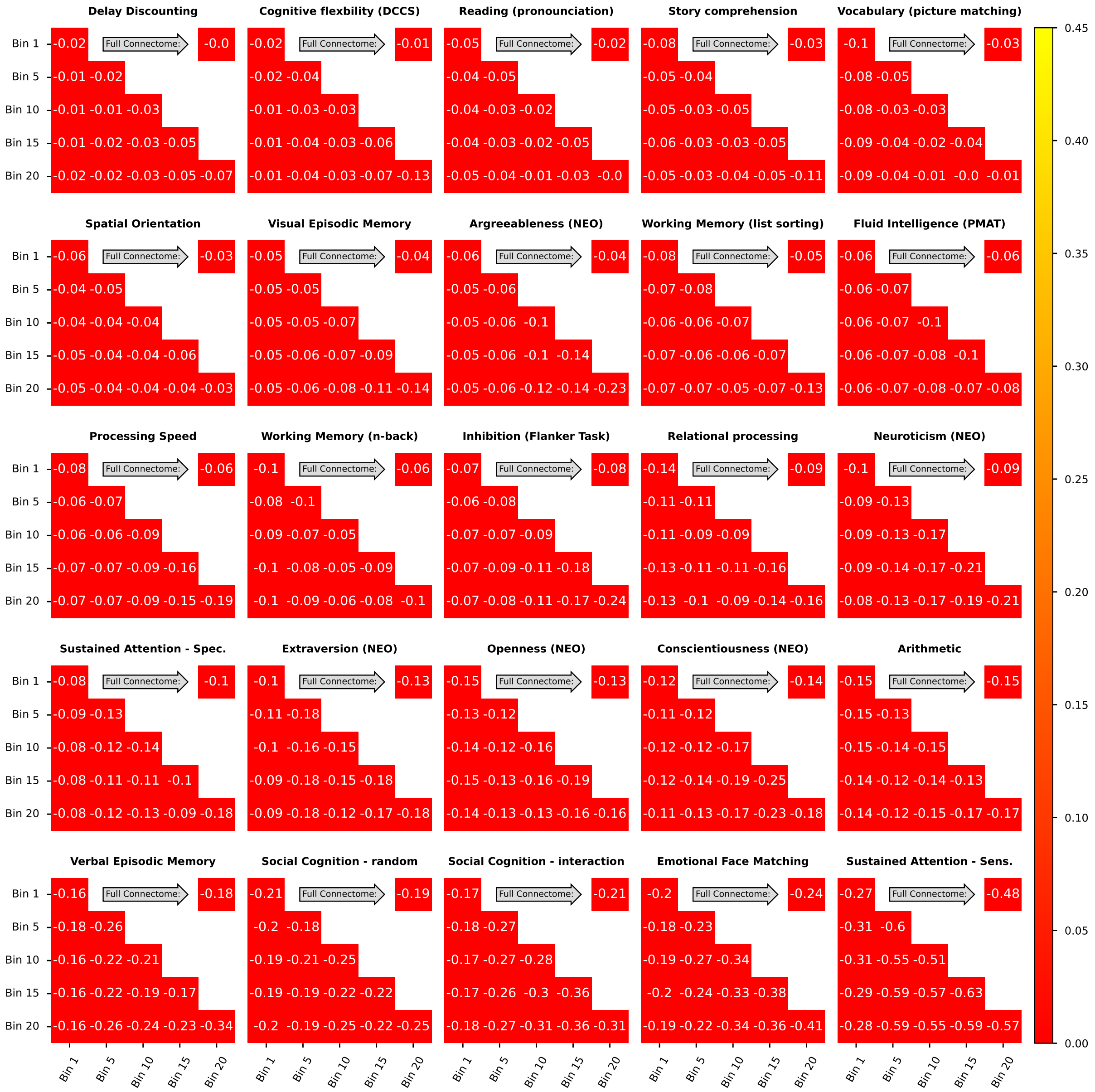

Supplementary Figure 7. Prediction scores (**r-squared**) in the **HCP-YA** sample for **Connectome-based Predictive Modeling (CBPM)** averaged across the ten folds in the grouped cross-validation scheme when using combined and individual bins sampling strategies and the **200 area Schaefer parcellation**. Scores for individual bins are displayed on the diagonal, for combined bins off the diagonal. Scores for the full FC using the whole time series are always displayed in the upper right corner.

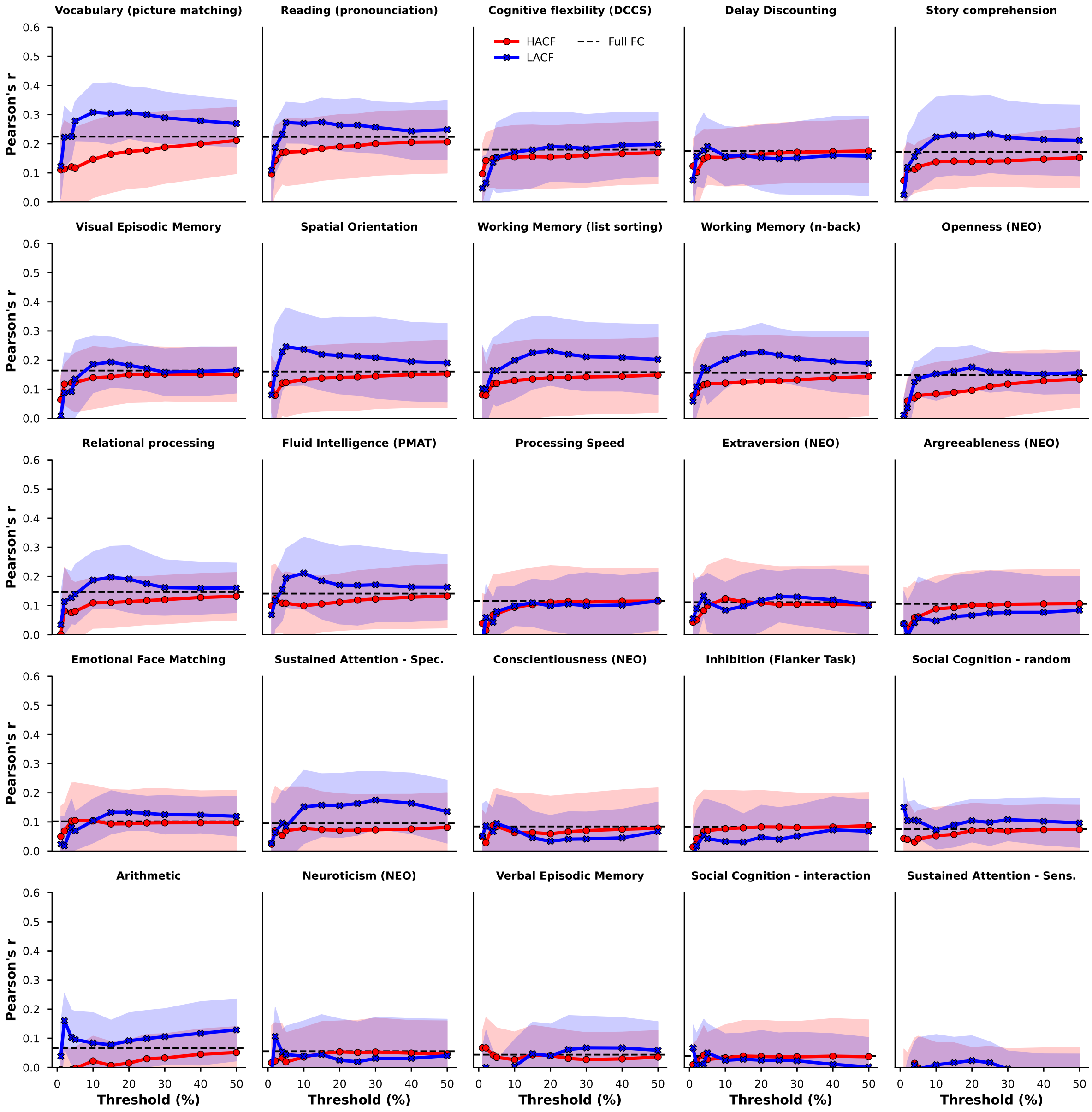

Supplementary Figure 8. Prediction scores (**Pearson's r** between observed and predicted values) in the **HCP-YA** sample for **CBPM** averaged across the ten folds in the grouped cross-validation scheme when using the **200 area Schaefer parcellation** and FC estimates derived from timepoints at different levels of co-fluctuation magnitude in the sequential sampling strategy.

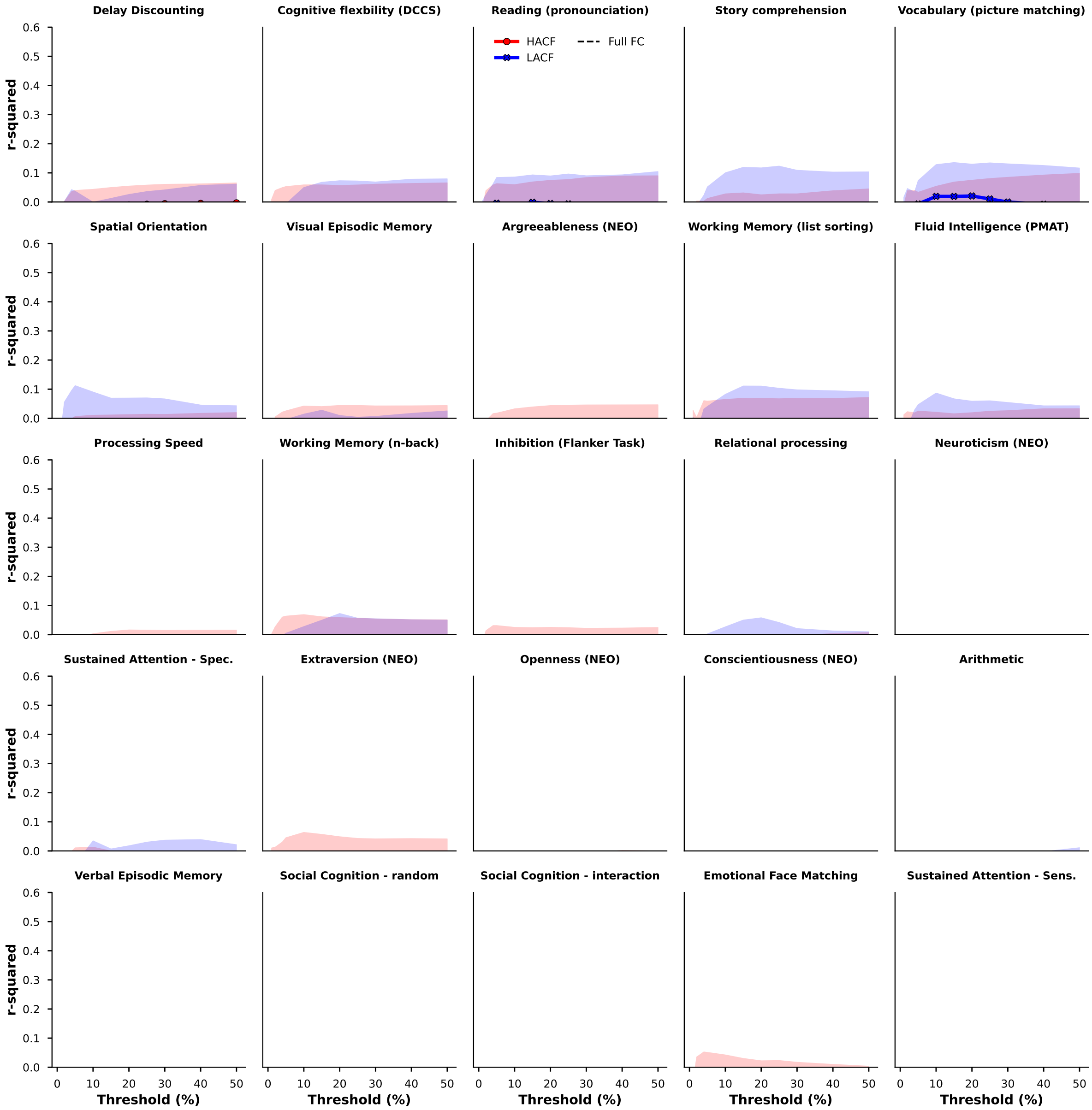

Supplementary Figure 9. Prediction scores (**r-squared**) in the **HCP-YA** sample for **CBPM** averaged across the ten folds in the grouped cross-validation scheme when using the **200 area Schaefer parcellation** and FC estimates derived from timepoints at different levels of co-fluctuation magnitude in the sequential sampling strategy.

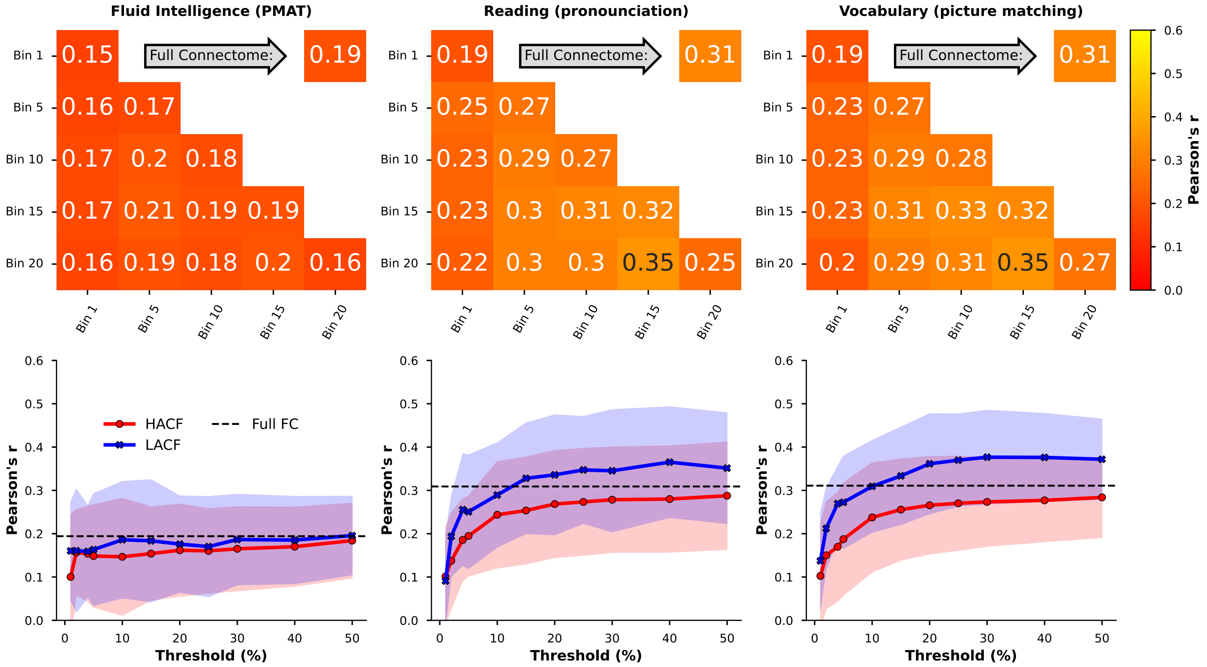

Supplementary Figure 10. Prediction scores (**Pearson's r** between observed and predicted values) in the **HCP-YA** sample for **kernel ridge regression** averaged across the ten folds in the grouped cross-validation scheme when using combined and individual bins sampling strategies and the **200 area Schaefer parcellation without global signal regression**

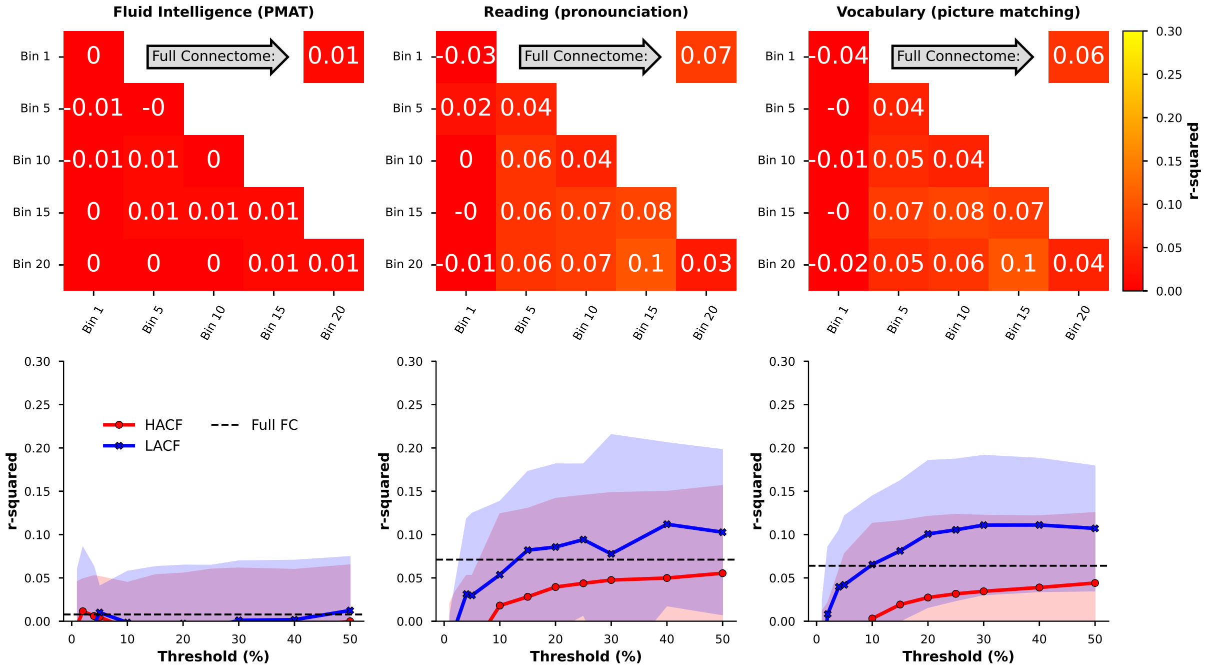

Supplementary Figure 11. Prediction scores (**r-squared** between observed and predicted values) in the **HCP-YA** sample for **kernel ridge regression** averaged across the ten folds in the grouped cross-validation scheme when using combined and individual bins sampling strategies and the **200 area Schaefer parcellation without global signal regression**

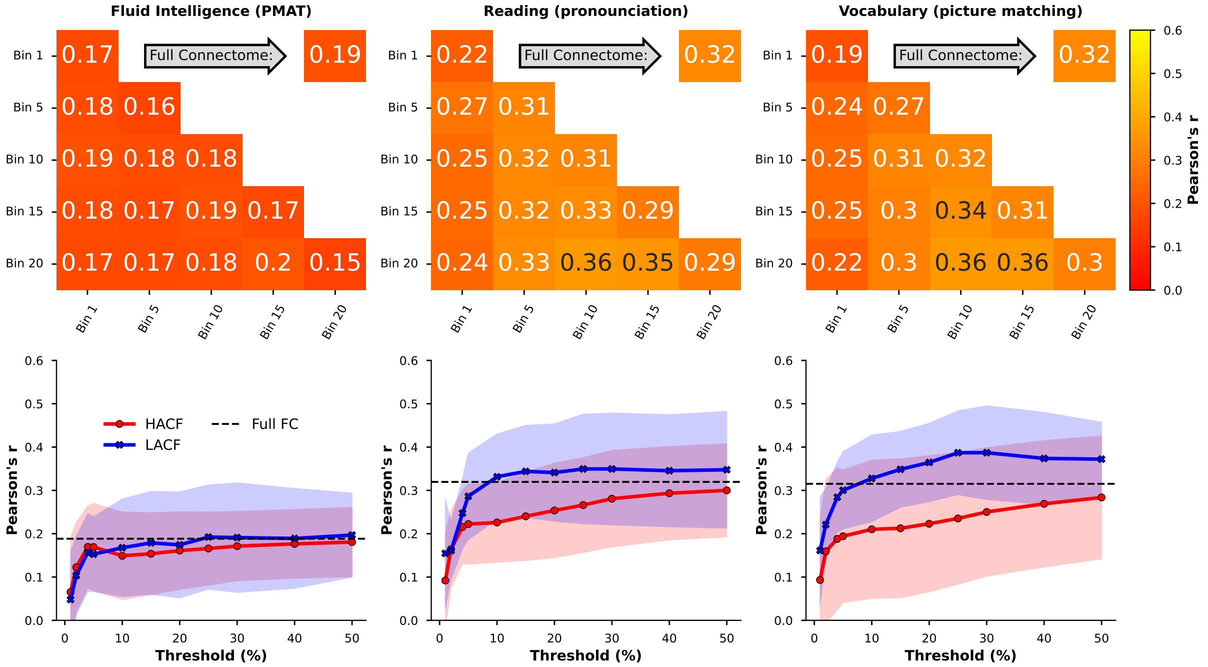

Supplementary Figure 12. Prediction scores (**Pearson's r** between observed and predicted values) in the **HCP-YA** sample for **kernel ridge regression** averaged across the ten folds in the grouped cross-validation scheme when using combined and individual bins sampling strategies and the **300 area Schaefer parcellation with global signal regression**

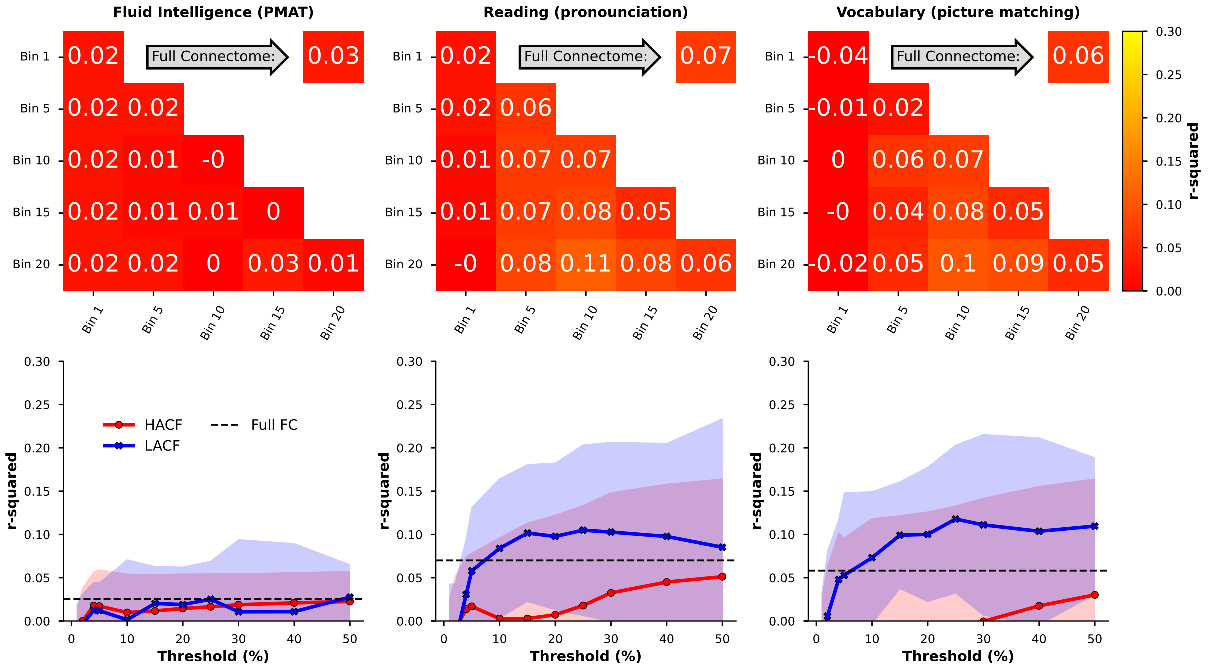

Supplementary Figure 13. Prediction scores (**r-squared** between observed and predicted values) in the **HCP-YA** sample for **kernel ridge regression** averaged across the ten folds in the grouped cross-validation scheme when using combined and individual bins sampling strategies and the **300 area Schaefer parcellation with global signal regression**

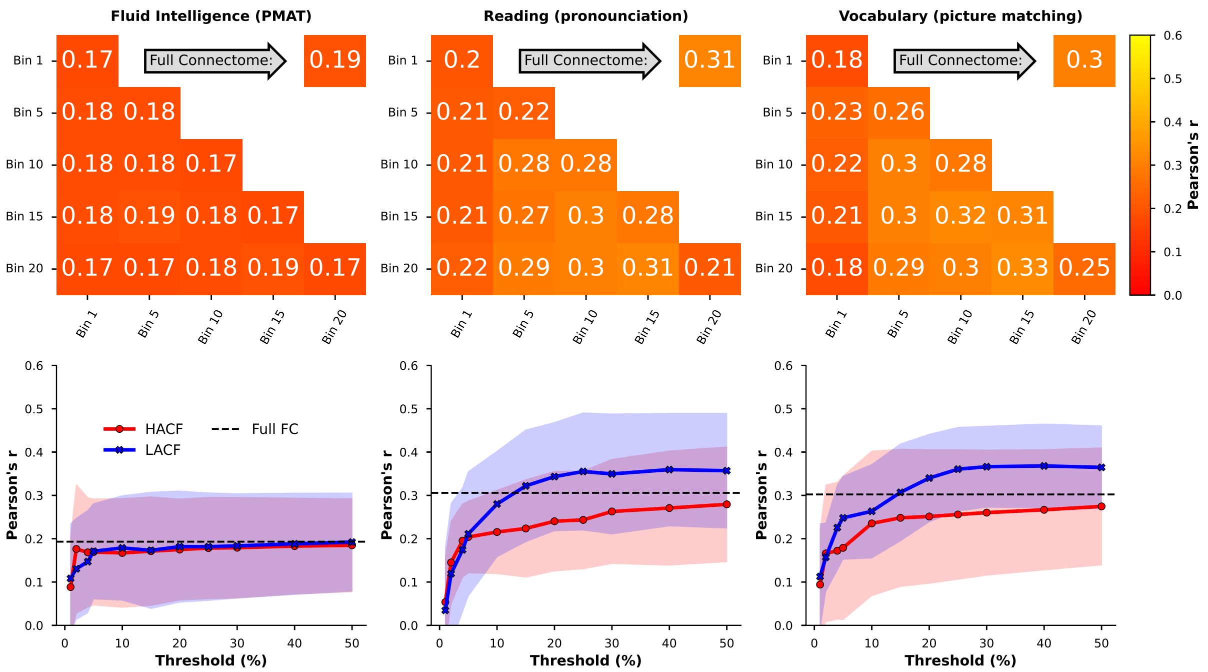

Supplementary Figure 14. Prediction scores (**Pearson's r** between observed and predicted values) in the **HCP-YA** sample for **kernel ridge regression** averaged across the ten folds in the grouped cross-validation scheme when using combined and individual bins sampling strategies and the **300 area Schaefer parcellation without global signal regression**

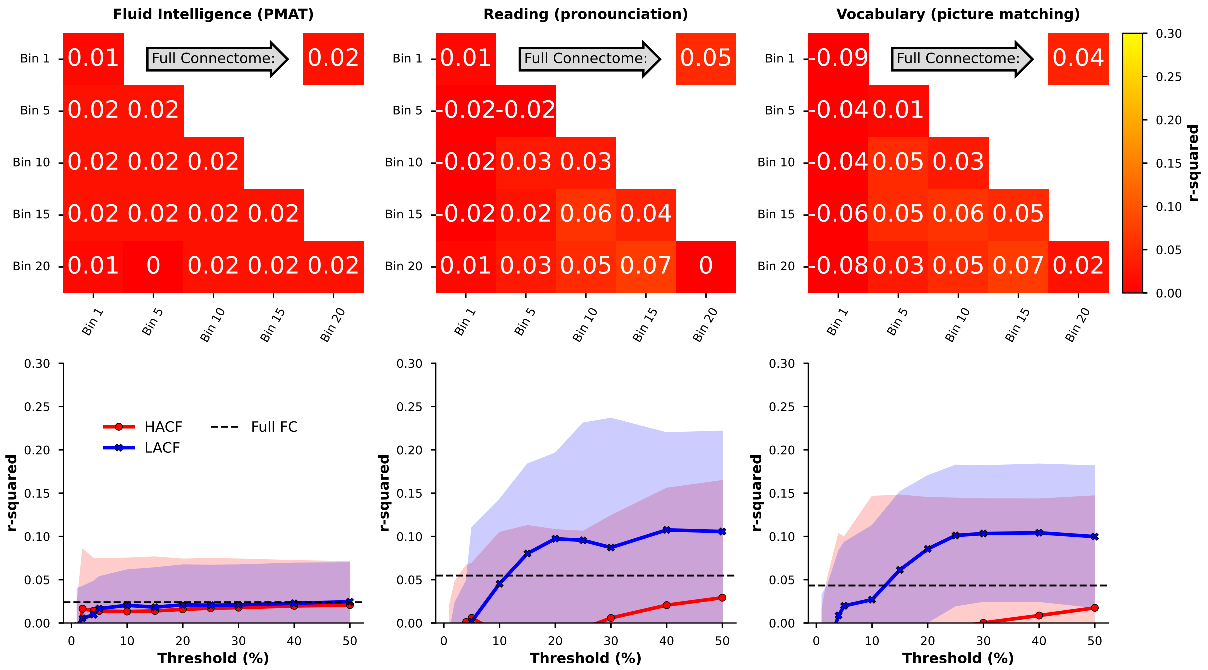

Supplementary Figure 15. Prediction scores (**r-squared** between observed and predicted values) in the **HCP-YA** sample for **kernel ridge regression** averaged across the ten folds in the grouped cross-validation scheme when using combined and individual bins sampling strategies and the **300 area Schaefer parcellation without global signal regression**

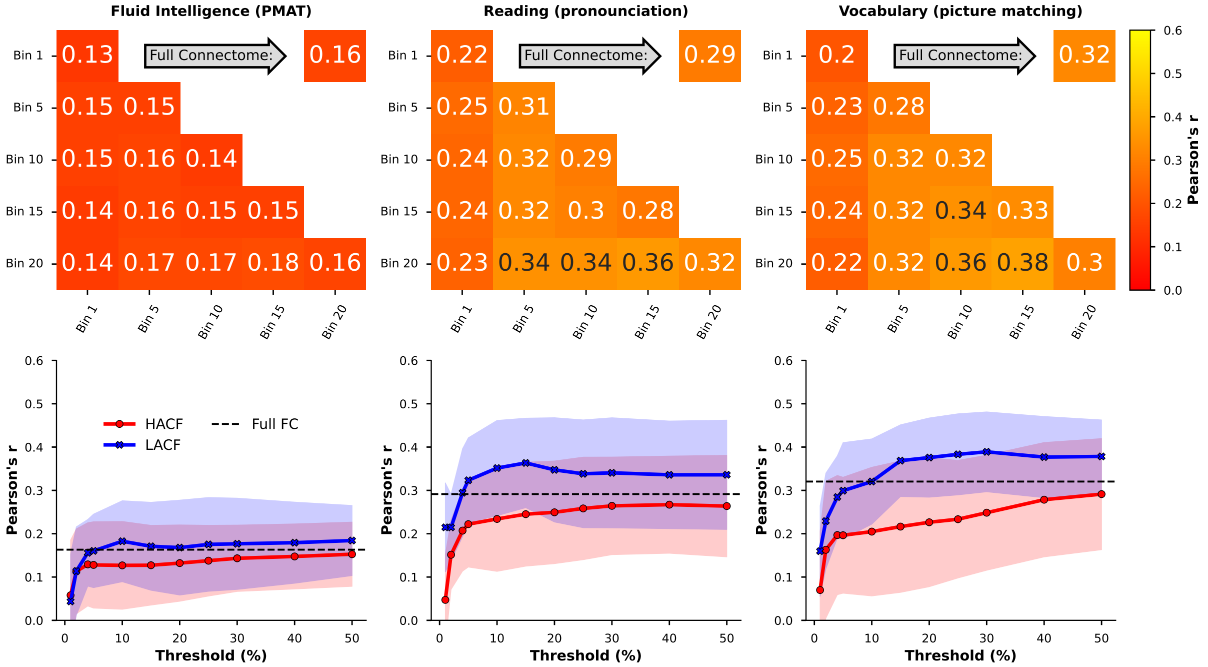

Supplementary Figure 16. Prediction scores (**Pearson's r** between observed and predicted values) in the **HCP-YA** sample for **kernel ridge regression** averaged across the ten folds in the grouped cross-validation scheme when using combined and individual bins sampling strategies and the **400 area Schaefer parcellation with global signal regression**

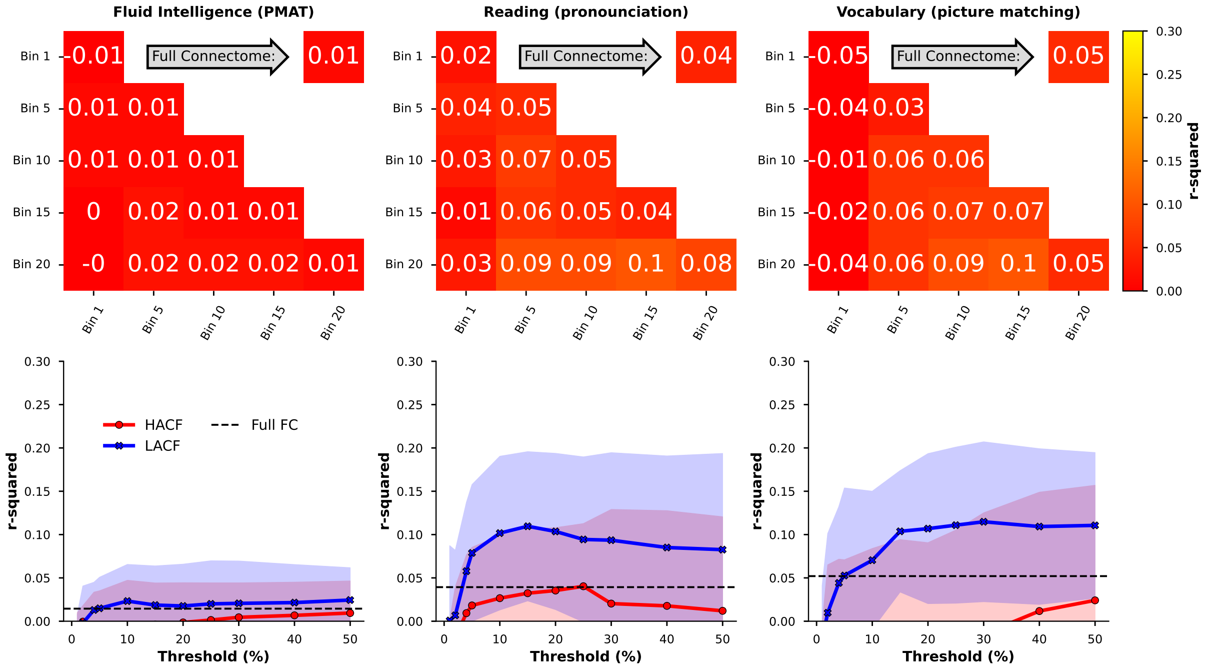

Supplementary Figure 17. Prediction scores (**r-squared** between observed and predicted values) in the **HCP-YA** sample for **kernel ridge regression** averaged across the ten folds in the grouped cross-validation scheme when using combined and individual bins sampling strategies and the **400 area Schaefer parcellation with global signal regression**

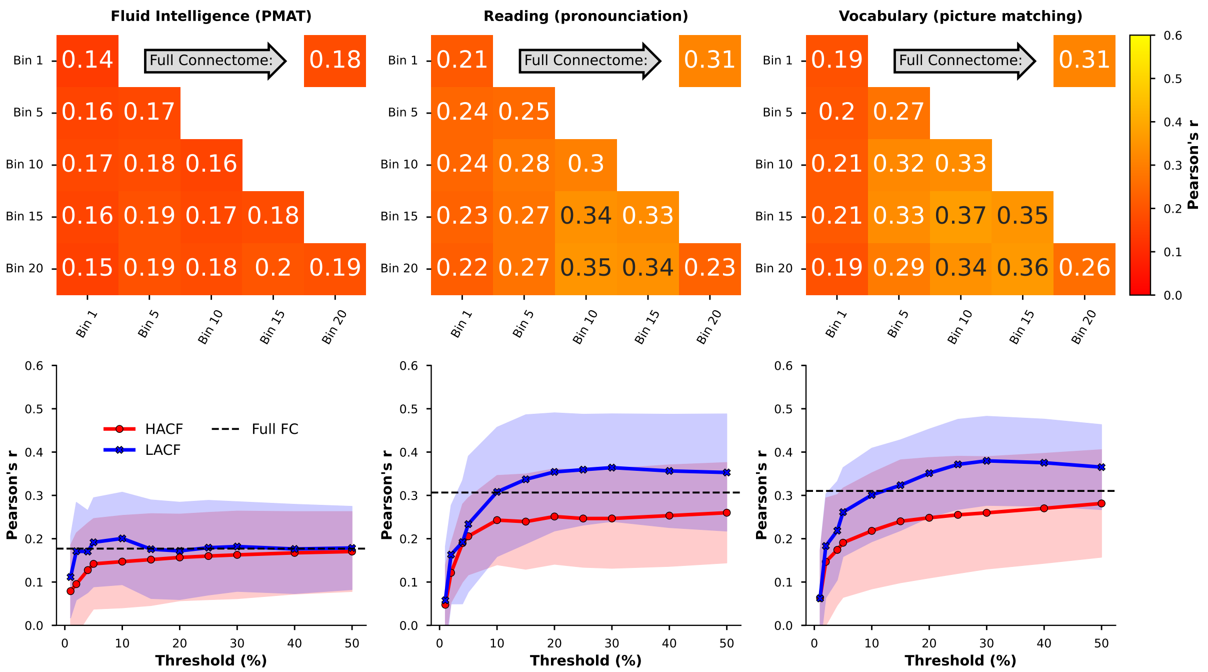

Supplementary Figure 18. Prediction scores (**Pearson's r** between observed and predicted values) in the **HCP-YA** sample for **kernel ridge regression** averaged across the ten folds in the grouped cross-validation scheme when using combined and individual bins sampling strategies and the **400 area Schaefer parcellation without global signal regression**

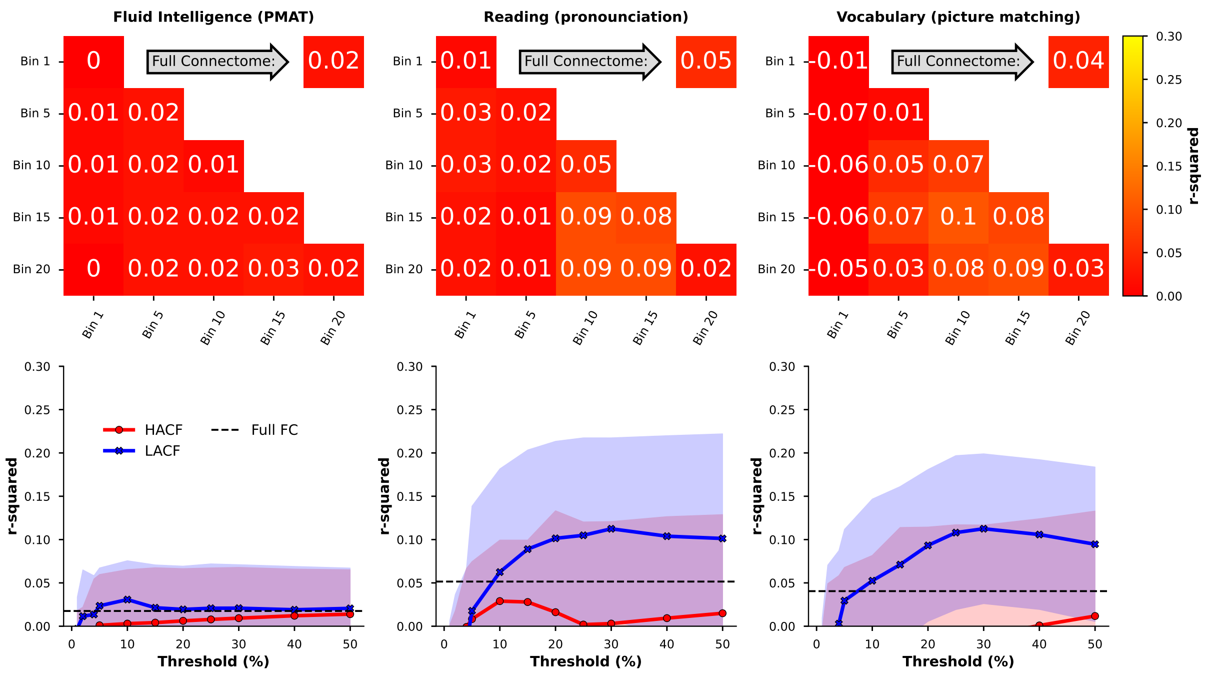

Supplementary Figure 19. Prediction scores (**r-squared** between observed and predicted values) in the **HCP-YA** sample for **kernel ridge regression** averaged across the ten folds in the grouped cross-validation scheme when using combined and individual bins sampling strategies and the **400 area Schaefer parcellation without global signal regression**

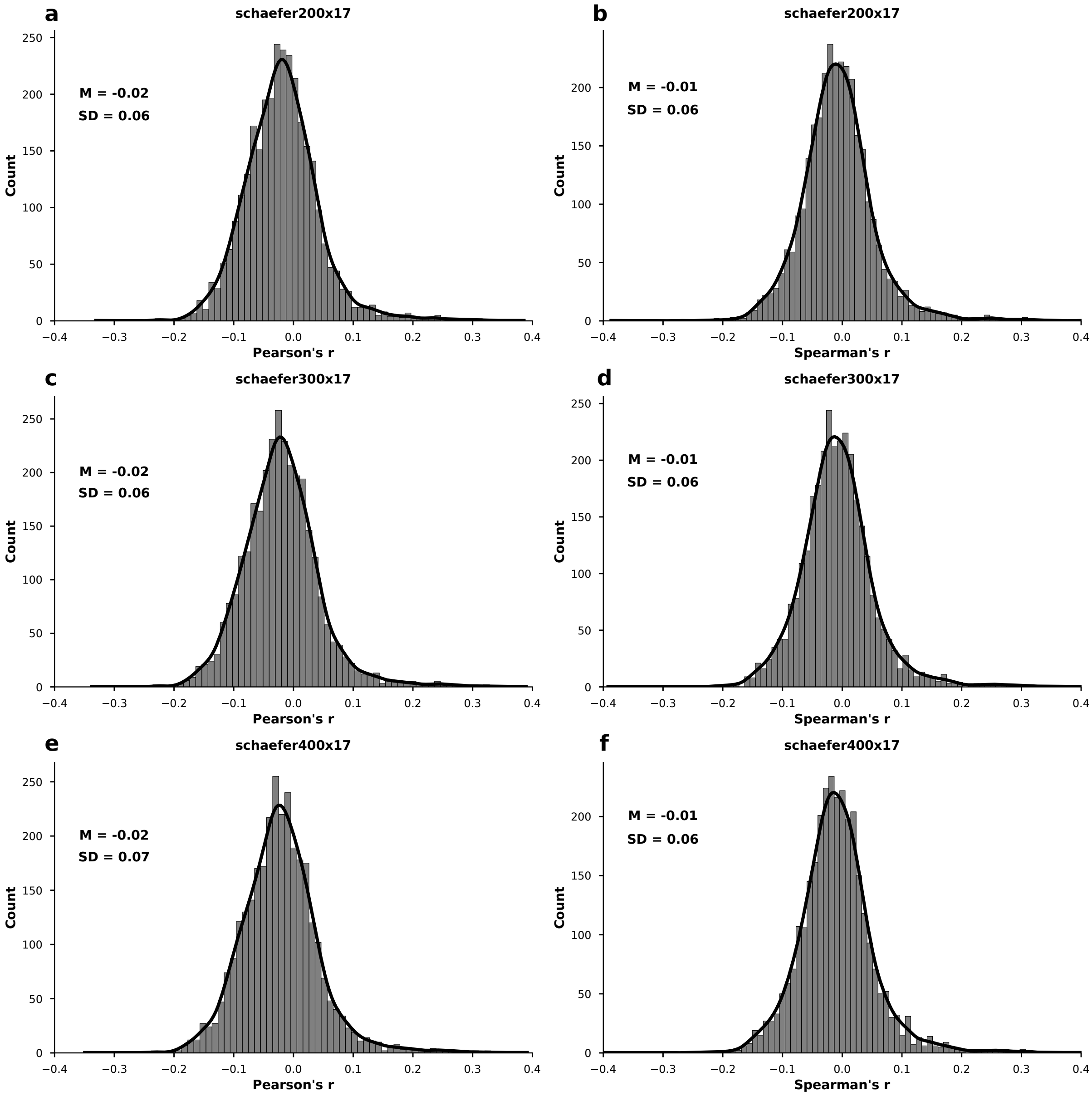

Supplementary Figure 20. Distribution of correlations between RSS and FD for every subject and every rs-fMRI run using three different parcellations and both Spearman's and Pearson's correlation coefficients.

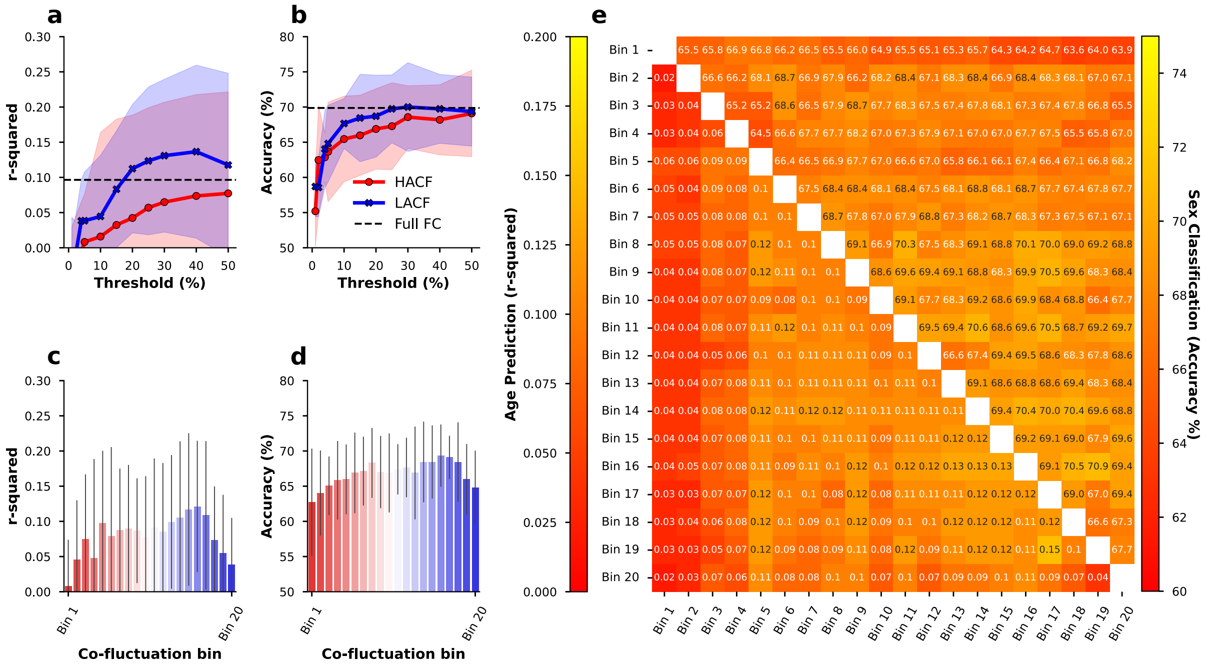

Supplementary Figure 21. Age prediction scores (**r-squared**) and sex classification accuracy in the **HCP-YA** sample for the sequential (**a** and **b**), individual bins (**c** and **d**) and the combined bins (**e**) sampling strategies. Here, in sex prediction we use a support vector classifier (SVC) with a linear kernel.

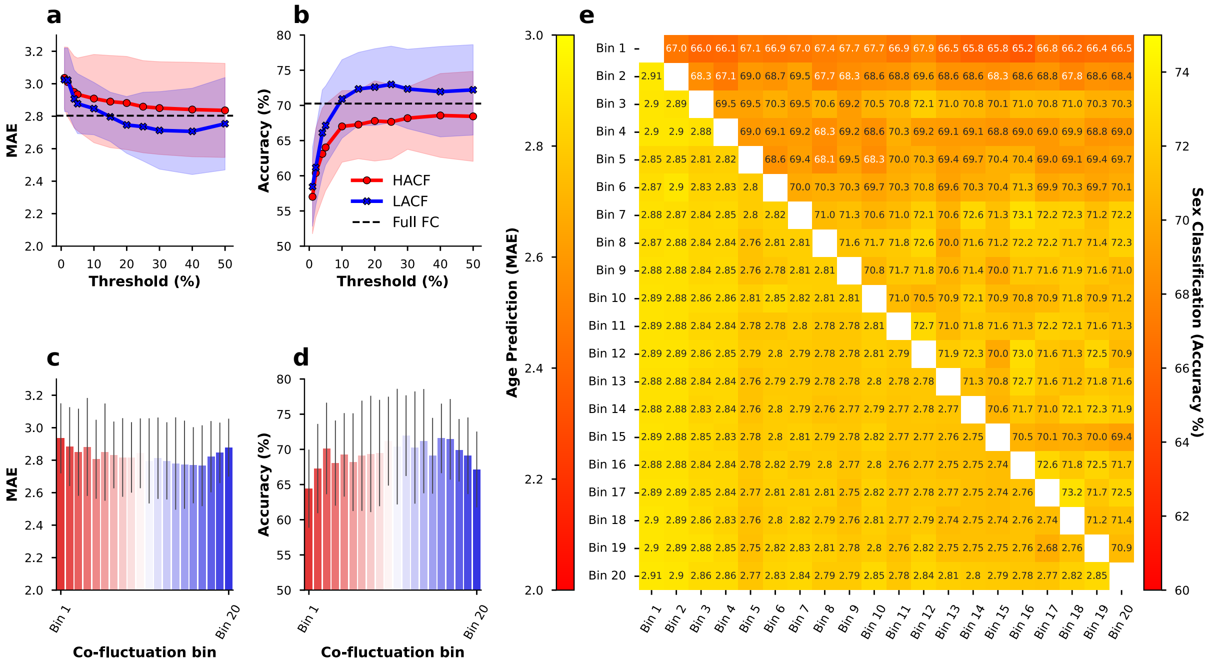

Supplementary Figure 22. Age prediction scores (**MAE**) and sex classification accuracy in the **HCP-YA** sample for the sequential (**a** and **b**), individual bins (**c** and **d**) and the combined bins (**e**) sampling strategies. Here, in sex prediction we use a SVC with a radial basis function (RBF) kernel.

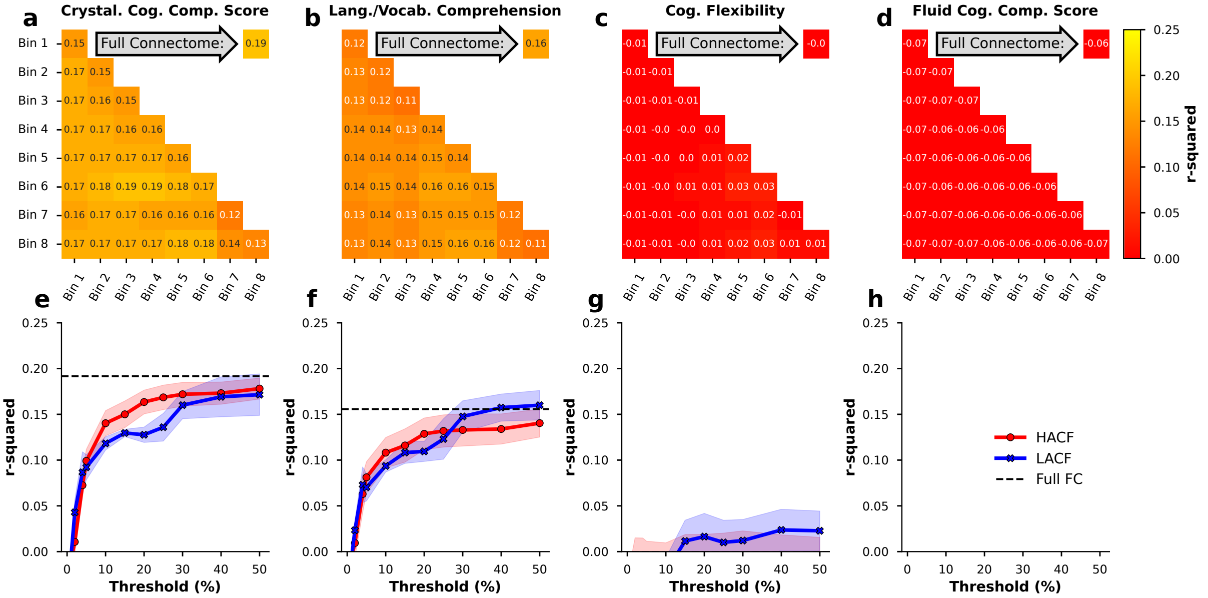

Supplementary Figure 23. Prediction accuracy (**r-squared**) for four cognitive targets in the **HCP-A** sample using the individual and combined bins strategy (**a-d**; individual bins are on the diagonal) and the sequential sampling strategy (**e-h**).

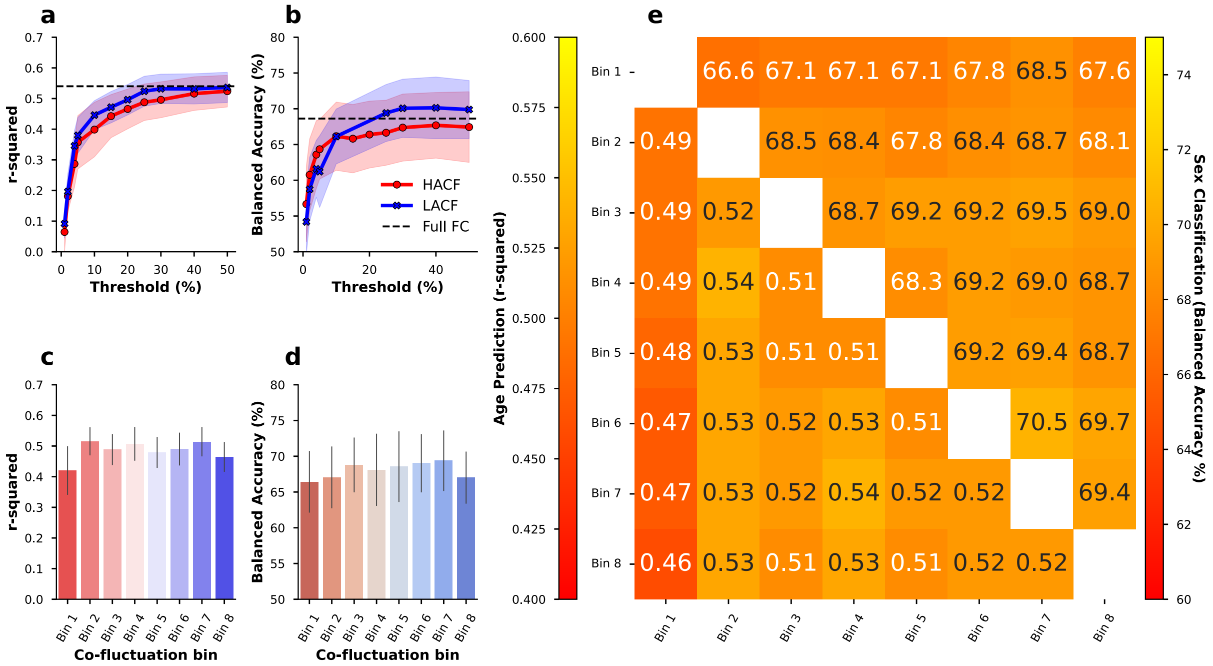

Supplementary Figure 24. Age prediction (**r-squared**) and sex classification (balanced accuracy) in the **HCP-A** sample using the sequential (**a** and **b**), individual bins (**c** and **d**), and combined bins (**e**) sampling strategies using the HCP-A sample. Here, in sex prediction we use a SVC with a linear kernel.

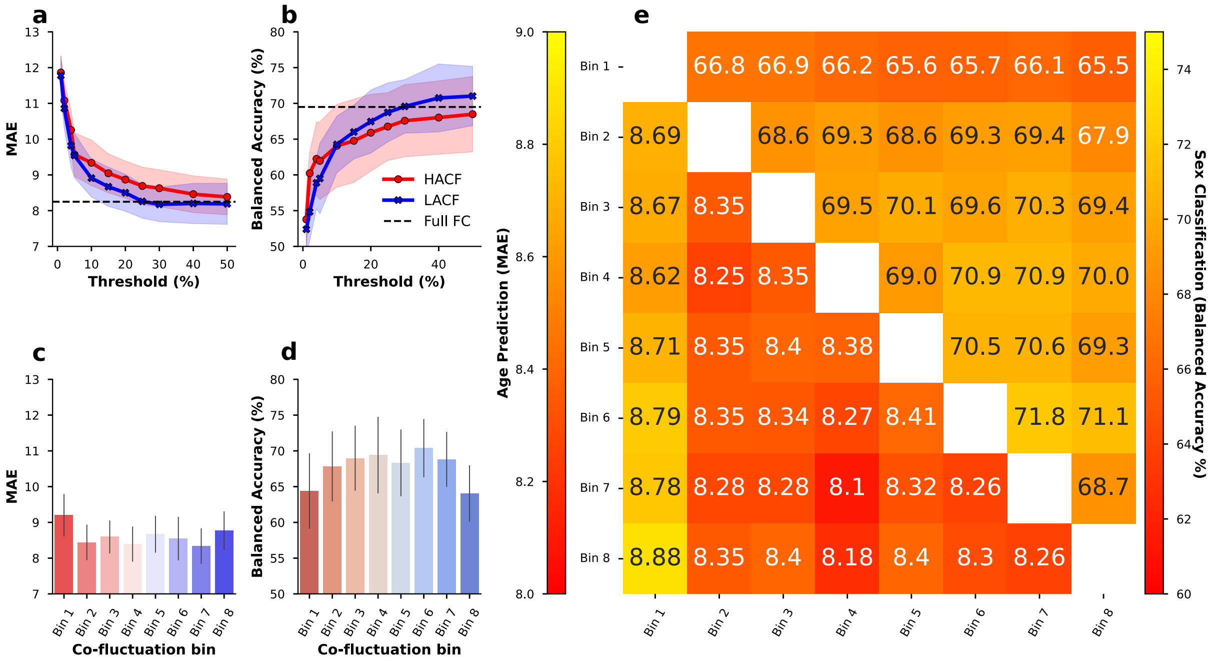

Supplementary Figure 25. Age prediction (**MAE**) and sex classification (balanced accuracy) in the **HCP-A** sample using the sequential (**a** and **b**), individual bins (**c** and **d**), and combined bins (**e**) sampling strategies using the HCP-A sample. Here, in sex prediction we use a SVC with a RBF kernel.

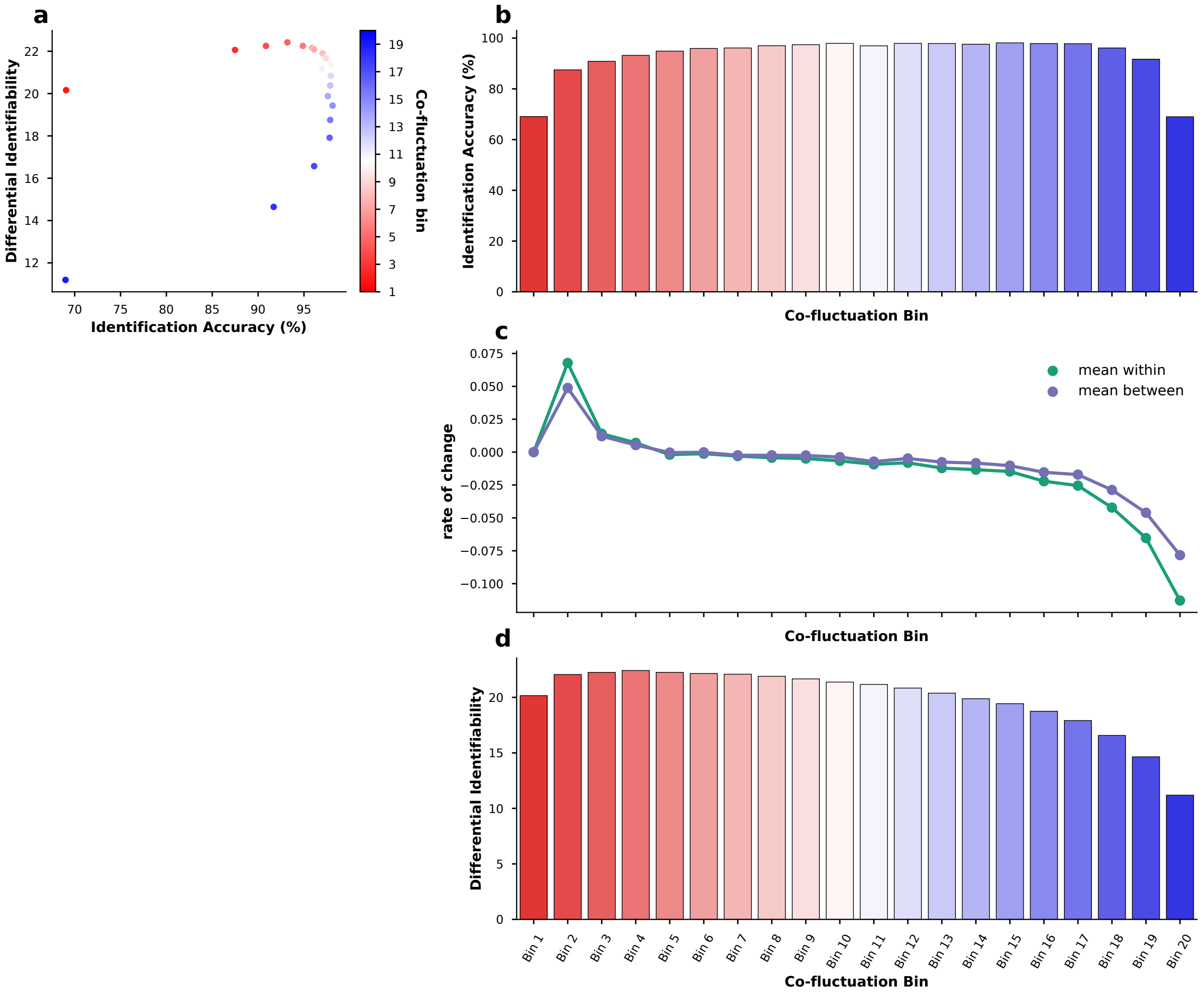

Supplementary Figure 26. Identification accuracy and differential identifiability in the HCP-YA sample using the individual bins strategy: **a)** shows optimal subject specificity of intermediate bins in a scatterplot of identification accuracy and differential identifiability. **b)** shows identification accuracy, **c)** shows the derivative (i.e. difference between one bin and the next) of mean within- and mean-between subject correlations and **d)** shows differential identifiability.

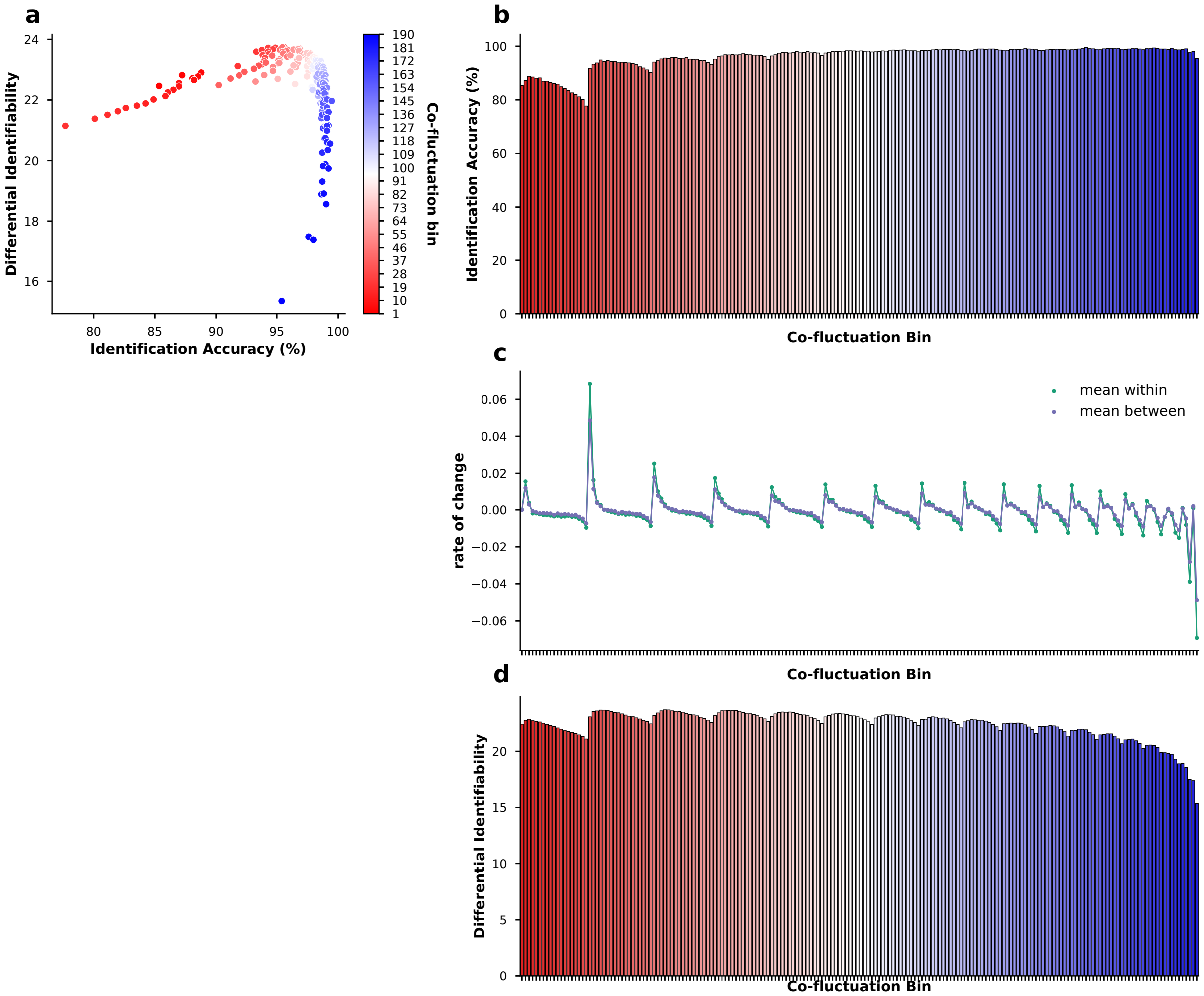

Supplementary Figure 27. Identification accuracy and differential identifiability in the HCP-YA sample using the individual bins strategy: **a)** shows optimal subject specificity of intermediate bins in a scatterplot of identification accuracy and differential identifiability. **b)** shows identification accuracy, **c)** shows the derivative (i.e. difference between one bin and the next) of mean within- and mean-between subject correlations and **d)** shows differential identifiability.

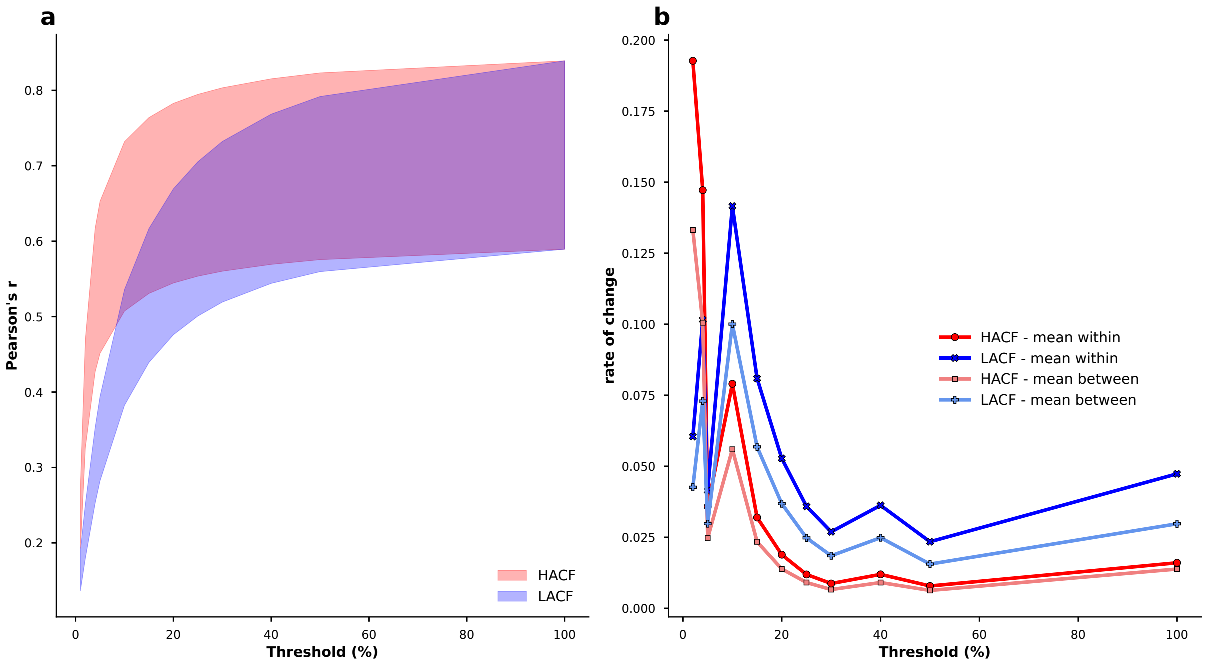

Supplementary Figure 28. **a)**: Within- and between-subject correlations in the sequential sampling strategy: fill colours indicate co-fluctuation level (i.e. “HACF” or “LACF”). The lower boundary of fill colour shows the mean between-subject correlation, whereas the upper boundary shows the mean within-subject correlation. **b)**: Derivative for each co-fluctuation level’s mean within- and between-subject correlations (i.e. rate of change from one threshold to the next).
