# Supplementary material for: Intermediately synchronised brain states optimise trade-off between subject specificity and predictive capacity
Source: Commun Biol. 2023 Jul 10;6:705. doi: 10.1038/s42003-023-05073-w (PMC10333234; doi:10.1038/s42003-023-05073-w)
Supplement: Supplementary file 3 — Description of Additional Supplementary Files [file 42003_2023_5073_MOESM3_ESM.pdf]

Description of Additional Supplementary Files

File name: Supplementary Data 1
Description: The results for age and sex prediction in both HCP-YA and HCP-AGING datasets. Contains source data for figures 4 and 6.

File name: Supplementary Data 2
Description: The results for identification analyses in both HCP-YA and HCP-AGING datasets. Contains source data for figures 1 and 5.

File name: Supplementary Data 3
Description: The results for all prediction analyses in which structural connectivity was regressed from functional connectivity. Contains source data for figure 8.

File name: Supplementary Data 4
Description: The results for prediction of psychometric variables in both HCP-YA and HCP-AGING datasets. Contains source data for figures 2, 3, and 6.

File name: Supplementary Data 5
Description: The results for analyses correlating structural and functional connectivity. Contains source data for figure 7.
