# Supplementary material for: Intermediately synchronised brain states optimise trade-off between subject specificity and predictive capacity
Source: Commun Biol. 2023 Jul 10;6:705. doi: 10.1038/s42003-023-05073-w (PMC10333234; doi:10.1038/s42003-023-05073-w)
Supplement: Supplementary file 9 — Reporting Summary [file 42003_2023_5073_MOESM9_ESM.pdf]

nature portfolio

Corresponding author(s): Leonard Sasse
Dr. Kaustubh Patil

Last updated by author(s): Jun 22, 2023

Reporting Summary

Nature Portfolio wishes to improve the reproducibility of the work that we publish. This form provides structure for consistency and transparency in reporting. For further information on Nature Portfolio policies, see our Editorial Policies and the Editorial Policy Checklist.

Statistics

For all statistical analyses, confirm that the following items are present in the figure legend, table legend, main text, or Methods section.

n/a	Confirmed	
☐	☒	The exact sample size (*n*) for each experimental group/condition, given as a discrete number and unit of measurement
☐	☒	A statement on whether measurements were taken from distinct samples or whether the same sample was measured repeatedly
☐	☒	The statistical test(s) used AND whether they are one- or two-sided<br>*Only common tests should be described solely by name; describe more complex techniques in the Methods section.*
☐	☒	A description of all covariates tested
☐	☒	A description of any assumptions or corrections, such as tests of normality and adjustment for multiple comparisons
☐	☒	A full description of the statistical parameters including central tendency (e.g. means) or other basic estimates (e.g. regression coefficient) AND variation (e.g. standard deviation) or associated estimates of uncertainty (e.g. confidence intervals)
☐	☒	For null hypothesis testing, the test statistic (e.g. *F*, *t*, *r*) with confidence intervals, effect sizes, degrees of freedom and *P* value noted<br>*Give P values as exact values whenever suitable.*
☐	☒	For Bayesian analysis, information on the choice of priors and Markov chain Monte Carlo settings
☐	☒	For hierarchical and complex designs, identification of the appropriate level for tests and full reporting of outcomes
☐	☒	Estimates of effect sizes (e.g. Cohen's *d*, Pearson's *r*), indicating how they were calculated

Our web collection on statistics for biologists contains articles on many of the points above.

Software and code

Policy information about availability of computer code

Data collection	For data retrieval, we used datalad (http://docs.datalad.org/en/stable/generated/datalad.api.Dataset.html) in combination with the HCP open-access datalad dataset (https://github.com/datalad-datasets/human-connectome-project-openaccess/tree/master/HCP1200).
Data analysis	Code used to generate edge time series, connectomes, and perform prediction and other analyses on these data can be found in a public GitHub repository (https://github.com/juaml/etspredict). The code used to obtain SC is available at https://jugit.fz-juelich.de/inm7/public/vbc-mri-pipeline.

For manuscripts utilizing custom algorithms or software that are central to the research but not yet described in published literature, software must be made available to editors and reviewers. We strongly encourage code deposition in a community repository (e.g. GitHub). See the Nature Portfolio guidelines for submitting code & software for further information.

Data

Policy information about availability of data

All manuscripts must include a data availability statement. This statement should provide the following information, where applicable:

- Accession codes, unique identifiers, or web links for publicly available datasets
- A description of any restrictions on data availability
- For clinical datasets or third party data, please ensure that the statement adheres to our policy

Further information on how to obtain the HCP-YA and the HCP-A datasets can be obtained at https: //www.humanconnectome.org/. Data and/or research tools used in the preparation of this manuscript were obtained from the National Institute of Mental Health (NIMH) Data Archive (NDA). NDA is a collaborative informatics system created by the National Institutes of Health to provide a national resource to support and accelerate research in mental health. Dataset identifier: [https://doi.org/10.15154/1527952]. This manuscript reflects the views of the authors and may not reflect the opinions or views of the NIH or of the Submitters submitting original data to NDA.

Human research participants

Policy information about studies involving human research participants and Sex and Gender in Research.

Reporting on sex and gender	HCP young adult dataset: 771 subjects (384 female, 387 male). Participants' age ranged from 22 to 37 (M=28.41, SD=3.74); HCP-Aging dataset: 558 subjects (316 female, 242 male) with ages ranging between 36 and 100 years (M=59.87, SD=15.03). Sex was used as a confounding variable for most prediction analyses.
Population characteristics	There were no further categorisations of population characteristics utilised in this study.
Recruitment	The subject recruitment was organised and carried out by the WU-Minn HCP consortium. The project primarily recruited healthy individuals born in Missouri. Efforts were taken to make sure the ethnic and racial composition broadly reflected the 2000 decennial census. Individuals with severe neurodevelopmental disorders, documented neuropsychiatric disorders, or neurological disorders were excluded.
Ethics oversight	The scanning protocol for both HCP-YA and HCP-A was approved by the local Institutional Review Board at Washington University in St. Louis. Retrospective analysis of these datasets was further approved by the local Ethics Committee at the Faculty of Medicine at Heinrich-Heine-University in Düsseldorf.

Note that full information on the approval of the study protocol must also be provided in the manuscript.

Field-specific reporting

Please select the one below that is the best fit for your research. If you are not sure, read the appropriate sections before making your selection.

☐ Life sciences ☒ Behavioural & social sciences ☐ Ecological, evolutionary & environmental sciences

For a reference copy of the document with all sections, see nature.com/documents/nr-reporting-summary-flat.pdf

Behavioural & social sciences study design

All studies must disclose on these points even when the disclosure is negative.

Study description	Here, we systematically evaluate brain-behaviour associations by assessing the predictive utility of functional connectivity estimates at different levels of co-fluctuation using machine learning (ML) approaches. We demonstrate that time points of lower and intermediate co-fluctuation levels provide overall highest subject specificity as well as highest predictive capacity of individual-level phenotypes.
Research sample	HCP young adult dataset: 771 subjects (384 female, 387 male). Participants' age ranged from 22 to 37 (M=28.41, SD=3.74); HCP-Aging dataset: 558 subjects (316 female, 242 male) with ages ranging between 36 and 100 years (M=59.87, SD=15.03).
Sampling strategy	This study only involved retrospective analyses only.
Data collection	Data was collected by the WU-Minn HCP Consortium and is available at https://db.humanconnectome.org/app/template/Login.vm.
Timing	Data collection ranged from 2012 to 2017.
Data exclusions	Subjects were excluded if they did not have all 4 resting-state fMRI scanning sessions, resulting in the sample above. For a few analyses further individual subjects had to be excluded. Notably in the analyses involving structural connectivity, only 762 subjects

	were used in the HCP young adult data, due to lack of diffusion weighted imaging data. Further, in the HCP young-adult dataset, one person (male) had to be excluded from the sex classification analysis due to lack of information on confounding variables.
Non-participation	None of the participants selected for this study dropped out retrospectively.
Randomization	Participants were not randomly allocated to experimental groups. In prediction analyses, confounding variables (commonly age, sex and head motion in scanner i.e. "framewise displacement" or "FD") were accounted for using cross-validation consistent linear regression based confound removal on the targets. In the case of sex classification, confounds were removed from the features (age, brain volume ["FS_BrainSeg_Vol"], educational status ["SSAGA_Educ"], and FD as confounds).

Reporting for specific materials, systems and methods

We require information from authors about some types of materials, experimental systems and methods used in many studies. Here, indicate whether each material, system or method listed is relevant to your study. If you are not sure if a list item applies to your research, read the appropriate section before selecting a response.

Materials & experimental systems

n/a	Involved in the study
☒	☐ Antibodies
☒	☐ Eukaryotic cell lines
☒	☐ Palaeontology and archaeology
☒	☐ Animals and other organisms
☒	☐ Clinical data
☒	☐ Dual use research of concern

Methods

n/a	Involved in the study
☒	☐ ChIP-seq
☒	☐ Flow cytometry
☐	☒ MRI-based neuroimaging

Magnetic resonance imaging

Experimental design

Design type	resting-state and diffusion weighted images
Design specifications	Resting-state data in the HCP young-adult dataset was collected on two distinct days. On each day scans were acquired for different phase encoding directions (left-to-right, right-to-left). Each session lasted ca. 15 minutes, resulting in 1 hour of resting state data. The diffusion weighted images in the HCP young-adult dataset were acquired with the following parameters: Siemens 3 T Verio, 2 mm isotropic voxels, 64 slices, 60 directions, 2 averages with reversed phase encoding polarity, b = 1500 s/mm ^ 2, TE/TR = 86/10,000 ms, GRAPPA = 2, scan time = 20 min. The HCP aging sample also consisted of 2 scanning sessions consisting of 2 runs each with opposite phase encoding directions (anterior-to-posterior and posterior-to-anterior). Each run lasted ca. 6:30 minutes resulting in ca. 26 minutes of resting state data per subject.
Behavioral performance measures	In the HCP young adult dataset we used 25 distinct phenotypic variables from the categories "Cognition", "In-scanner task performance" and "personality". An exact list of these variables is outlined in Table 1 of the supplementary material. In the HCP-aging dataset we used 4 behavioural measures ("nih_crycogcomp_unadjusted", "tpvt_uss", "nih_dccs_unadjusted", and "nih_fluidcogcomp_unadjusted").

Acquisition

Imaging type(s)	rs-fMRI, diffusion weighted MRI
Field strength	3T
Sequence & imaging parameters	rs-fMRI data in HCP young-adult: 2D multiband gradient echo-planar imaging sequence with TE=33.1ms, TR=720ms, flip angle = 52°, 2.0mm isotropic voxels, 72 slices, multiband factor of 8). rs-fMRI in the HCP-aging dataset: 2D multiband (MB) gradient-recalled echo (GRE) echo-planar imaging (EPI) sequence (MB8, TR/TE = 800/37 ms, flip angle = 52°) and 2.0 mm isotropic voxels covering the whole brain (72 oblique-axial slices).
Area of acquisition	whole brain

Diffusion MRI ☒ Used ☐ Not used

Parameters	Diffusion weighted images (DWI) in HCP young-adult dataset: 2 mm isotropic voxels, 64 slices, 60 directions, 2 averages with reversed phase encoding polarity, b = 1500 s/mm ^ 2, TE/TR = 86/10,000 ms, GRAPPA = 2, scan time = 20 min. DWI images were preprocessed using the HCP diffusion minimal preprocessing pipeline. Further preprocessing and structural connectivity extraction were carried out using an in-house software tool available at https://jugit.fz-juelich.de/inm7/public/vbc-mri-pipeline.

Preprocessing

Preprocessing software	In both HCP young-adult and aging datasets, fMRI was preprocessed by the HCP using the HCP preprocessing pipeline and ICA+FIX denoising strategies. Subsequent preprocessing and feature extraction was performed using python and relevant third party libraries including nilearn. Code for all preprocessing can be found at https://github.com/juaml/etspredict. DWI images were preprocessed using the HCP diffusion minimal preprocessing pipeline. Further preprocessing and structural connectivity extraction were carried out using an in-house software tool available at https://jugit.fz-juelich.de/inm7/public/vbc-mri-pipeline.
Normalization	non-linear (FNIRT) registration into MNI152 space, grand-mean intensity normalisation
Normalization template	MNI152 space
Noise and artifact removal	In both datasets ICA+FIX denoising strategy was applied and motion parameters as well as their squared terms and derivatives were regressed out. In addition, we regressed out confounds, linearly detrended and bandpass filtered the signal at 0.008 - 0.08 Hz using “nilearn.image.clean_img”. This included mean time courses of the white matter (WM), cerebro-spinal fluid (CSF), and global signal (GS), as well as their squared terms and temporal derivatives as confounds. A spike regressor was further added for each fMRI frame exceeding a motion threshold (0.25 mm root mean squared framewise displacement).
Volume censoring	No volume censoring was carried out.

Statistical modeling & inference

Model type and settings	Predictive models were used to predict phenotypes from functional connectivity.
Effect(s) tested	A number of metrics were used to assess goodness of predictions (Pearson's r, R-squared, as well as mean absolute error in case of regression and accuracy as well as balanced accuracy in the case of classification).

Specify type of analysis: ☒ Whole brain ☐ ROI-based ☐ Both

Statistic type for inference (See Eklund et al. 2016)	Test scores were compared using the Bayesian region-of-practical-equivalence approach.
Correction	No correction for multiple comparisons undertaken.

Models & analysis

n/a	Involved in the study
☐	☒ Functional and/or effective connectivity
☒	☐ Graph analysis
☐	☒ Multivariate modeling or predictive analysis

Functional and/or effective connectivity	Functional connectivity was estimated using the Pearson's correlation coefficient.
Multivariate modeling and predictive analysis	Machine learning models were used to predict phenotypes from functional connectivity. We used kernel ridge regression, connectome-based predictive modelling, a ridge classifier, and a support vector classifier with both linear and radial basis function kernels.
